# The vulnerability of refugees and asylum seekers in Italy: Insights from a nationwide survey

**DOI:** 10.1371/journal.pone.0341950

**Published:** 2026-03-25

**Authors:** Daria Mendola, Eralba Cela, Livia Elisa Ortensi, Maurizio Ambrosini, Micaela Arcaio, Elisa Barbiano di Belgiojoso, Annalisa Busetta, Roberto Impicciatore, Eleonora Miaci, Marta Parigi, Anna Maria Parroco, Manuela Stranges, Francesca Tosi

**Affiliations:** 1 Department of Psychology, Educational Science and Human Movement, University of Palermo, Palermo, Italy; 2 Department of Social and Political Sciences, University of Milan, Milan, Italy; 3 Department of Statistical Sciences, Alma Mater Studiorum – University of Bologna, Bologna, Italy; 4 Department of Statistics and Quantitative Methods, University of Milano-Bicocca, Milan, Italy; 5 Department of Economic, Business, and Statistical Sciences, University of Palermo, Palermo, Italy; 6 Department of Economics, Statistics and Finance “Giovanni Anania”, University of Calabria, Arcavacata di Rende, Italy; El Colegio de la Frontera Norte, MEXICO

## Abstract

Despite the high number of news articles, images and public debate on forcibly displaced individuals, there is, with some rare exceptions, a lack of comprehensive surveys on their living conditions. In this context, our paper contributes to filling part of this gap by presenting the results of a new survey conducted in Italy in 2024 in the framework of the AVRAI research program. The survey collected responses from 1,327 adults with international protection or a history of seeking asylum, who arrived in Italy after 2011. This paper describes the main dimensions of vulnerability, offering insights on existing disparities based on gender and area of origin. Our survey reveals legal uncertainty, especially common among newcomers from Bangladesh, Pakistan and MENA countries. Health data shows that self-rated physical health is generally good but mental health outcomes are poorer, particularly among women, recent arrivals and those from Central and the Horn of Africa. Women also face greater employment challenges. Economic hardship is widespread, with over one-third experiencing severe material deprivation and high food insecurity, especially among Nigerians, Sub-Saharan Africans and MENA nationals. Despite adversities, many respondents show strong resilience, particularly those with higher education and coming from Sub-Saharan Africa and the Horn of Africa. About two thirds of respondents plan to remain in Italy and nearly two thirds feel welcome in Italy. However, discrimination and racism, especially against individuals of African origin, remain a significant concern. Although many express their satisfaction with life in Italy, experiences of exclusion are common. This survey sheds light on legal precarity, health risks, economic vulnerability, living conditions and lived experiences and perceptions in the peculiar Italian political, legal and administrative environment.

## Introduction

According to the last available data [1], as of the end of June 2024, 122.6 million people are fleeing around the world, escaping from persecution, war and conflicts in their own countries. These migrants are typically refereed as “Forcibly displaced persons”, an umbrella term which includes [1]:

- Refugees, “people who have fled their countries to escape conflict, violence, or persecution and are seeking safety in another country and are entitled to international protection”
- Asylum seekers, “someone who is seeking international protection. Their request for refugee status, or complementary protection status, has yet to be processed, or they may not yet have requested asylum but they intend to do so.”
- Internally displaced people – those who “have been forced to flee their homes by conflict, violence, persecution or disasters, however, unlike refugees, they remain within their own country”.

Refugee status is granted within the framework established by the 1951 Geneva Convention and the 1967 Protocol, whereas other forms of international protection may be conferred under national legislation for migrants in particularly vulnerable circumstances. In academic literature, individuals recognised as refugees and those granted other forms of international protection are often treated as a single analytical category (shortly labelled “refugees”), reflecting the common drivers and experiences underlying their displacement. This grouping yields a relatively homogeneous population that is clearly distinguishable from asylum seekers, whose situation is characterised by greater precarity and by the uncertainty surrounding the adjudication of their legal protection status and the extent of their entitlements. The number of forcibly displaced persons worldwide has increased by over 5 million in the last six months of 2024 and is approximately three times higher than it was 20 years before. Among these forcibly displaced persons, 43.7 million are refugees, 8 million are asylum-seekers and 72.1 million are internally displaced people. The share of those reaching high-income countries is around 29%, while 71% are hosted in low- and middle-income countries (69% hosted in neighbouring countries) [1].

In 2024, European Union countries received over 911 thousand first-time applications for international protection from non-EU citizens, with Italy being the third in Europe as a destination country for asylum seekers. Over the past decade, the number of asylum seekers in Italy has fluctuated, with periods of significant increase, followed by a decline, and in 2024, a gradual rise in the number of applicants [2].

The influx of immigrants and asylum seekers into Europe has posed a significant challenge for European leaders and policymakers in recent years. Immigration has emerged as a highly politicised issue, sparking polarisation and political tensions both within and between European nations. This situation has highlighted the difficulty of balancing humanitarian responsibilities with security concerns and intensified debates over national identity, cultural integration and economic impact. While some argue that immigrants contribute to the labour force and economic growth, others raise concerns about social cohesion, the strain on public services and the rise of far-right populism. Furthermore, the European Union’s response has been fragmented, with differing policies and approaches across member states, complicating efforts to find a unified solution to the crisis [3–5].

Despite the importance of the topic, a critical gap in addressing the challenges posed by the increase in refugee and asylum seeker flows is the lack of comprehensive data adequate to capture the unique circumstances of these populations. While general migration and demographic surveys often include refugees, beneficiaries of other forms of international protection and asylum seekers as part of a broader sample, these surveys typically do not delve deeply into the specific challenges, needs, or experiences of these groups [6]. Refugees and asylum seekers face distinct barriers, also with respect to other migrants, including legal uncertainties, trauma and cultural adjustment, which are not always reflected in standard surveys. Without dedicated, targeted surveys focusing on their experience, critical issues such as access to healthcare, mental health needs, language acquisition, integration into the labour market, traumatic migration experiences and asylum procedures, remain under-explored [7]. This lack of specialised data leads to an incomplete understanding of the integration of beneficiaries of international protection and asylum seekers, making it harder for policymakers to create responsive, evidence-based strategies that can address the full range of their needs.

Furthermore, only a limited number of countries have implemented longitudinal or panel studies specifically targeted at these populations, such as the IAB-BAMF-SOEP Refugee Survey in Germany [8] and the Survey of New Refugees, 2005–2009 in the UK [9]. These datasets provide valuable insights into employment trajectories, education, health and integration outcomes, showing the advantages of microdata in capturing the dynamics of beneficiaries of international protection and asylum seekers’ inclusion and exclusion over time. Other examples, but on a smaller scale than that of the German survey and lacking the longitudinal dimension, are those in Austria (namely, the Austrian Refugee Health and Integration Survey – ReHIS – and the DiPAS –Displaced Persons in Austria Survey).

The relatively small number of beneficiaries of international protection and asylum seekers in Italy (as of 2024, there are 312,849 refugees under UNHCR mandate and 207,278 asylum seekers in Italy [1]) combined with the practical challenges of identifying and accessing this specific population, the increasingly outdated perception of Italy as merely a transit country and the frequent conflation of asylum seekers, refugees and other beneficiaries of internation protection with migrants, may help explain the widespread lack of knowledge regarding the characteristics of asylum seekers and refugees living in Italy, as well as the absence of comprehensive periodic official surveys on this population.

Indeed, there have been some efforts of targeted surveys in Italy – such as the “*Condizione e Integrazione Sociale dei Cittadini Stranieri*” (i.e., Condition and integration of foreign citizens) by ISTAT [10] and survey-based work by Fondazione ISMU – which include refugee sub-samples. More recently, a World Bank and CeSPI study [11] focused on the educational and social integration of Ukrainian refugee minors and their caregivers in Italy, collecting detailed survey data on their school experiences and support needs.

However, these data sources are often limited in scope, lack continuity and rarely disaggregate findings by legal status or migration trajectory. This absence of granular, cross-sectional or longitudinal evidence makes it difficult to assess vulnerability across key dimensions of integration – such as legal uncertainty, housing stability, mental and physical health and access to training or employment– identified as central by Ager and Strang [6]. Moreover, recent research highlights how temporary or insecure legal status can severely constrain integration outcomes across these domains, further underscoring the need for targeted, evidence-based policies [12].

The lack of proper data sources in Italy directly reflects the scarcity of empirical studies. Using administrative microdata from a reception centre, Stranges and Wolff [13] contribute by examining the trajectories of asylum seekers and other informal migrants who pass through the reception system in Italy, though their study does not fully explore broader dimensions of vulnerability such as housing, healthcare access, or mental well-being. Some field-based qualitative studies have contributed some insights on these aspects. For instance, Zamperini et al. [14] conducted life-history interviews with Afghan refugees in Italy in 2021, highlighting themes of trauma, resilience and social adaptation. Bianco and Ortiz Cobo [15] examined linguistic integration through ethnographic research with adult refugees and Italian language teachers, revealing the need for more tailored and accessible language programs. Belloni, in her studies on the Eritrean communities in Rome (e.g., [16]), focused on the living conditions of this community in occupied buildings, highlighting the positive effects for refugees of joining fellow citizens who act as facilitators in accessing crucial economic and social resources and in sharing information about the Italian bureaucratic system and lifestyle, which helps foster integration.

A strand of studies on asylum seekers and refugees, living in informal settlements across Italy, is grounded on quantitative microdata collected via a probability field survey in 2016 [17,18]. These studies offer a comprehensive, quantitative and multidimensional perspective on their vulnerability, health and living conditions. In Mendola and Busetta [18], a national, although small-sized, survey highlights that many asylum seekers and refugees live in precarious housing conditions with limited access to essential services such as water, electricity and healthcare. In another study on the same population living in informal settlements, it is shown that social networks play a protective role, with those lacking family support being more likely to report poor health outcomes [19]. Furthermore, latent trait analysis is used to provide a rigorous approach for assessing vulnerability, revealing significant regional disparities and greater vulnerability among refugees and asylum seekers from Asia compared to those from Africa [20].

This article presents and discusses the descriptive results of a new field survey – *ItRAS*, Italian Refugees and Asylum Seekers Survey – targeting specifically asylum seekers with pending requests, recognised refugees, beneficiaries of other forms of international protection and Italian citizens with previous asylum-seeking background. For the sake of conciseness, this composite target population will hereafter be referred to collectively as “asylum seekers and refugees” or by the acronym “AS&Rs” used, hereafter, only when referring to the *ItRAS* target population. *ItRAS* was conducted in Italy in 2024 in the framework of the national research program AVRAI (Assessing Vulnerability of Refugees and Asylum Seekers in Italy). The survey outlines the socio-demographic and migration profiles of this target population, their relations with the legal context in Italy and their experiences and perceptions regarding integration in Italy. By shedding light on these aspects, the research project contributes to a better understanding of the lived experiences, vulnerabilities and strengths of individuals involved in the asylum process, aiming to expand knowledge of this under-investigated population in Italy. This paper only aims at presenting the survey and its descriptive findings, while other outputs of the project will build on these findings.

The remainder of the paper is structured as follows: The introduction concludes by providing a context to the paper, sizing the phenomenon in Italy with reference to the European Union and introducing the rules for granting international protection and the critical issues of the Italian national procedures; the “Material and Methods” section is devoted to the presentation of the *ItRAS* (Italian Refugees and Asylum seekers’ survey), its questionnaire, the sampling technique, the fieldwork organization, the adopted weights and the structure of the sample; the section “Results” presents and discusses the descriptive findings from the survey regarding some of the investigated dimensions (legal sphere, physical and mental health, resilience, food security, labour market integration, material deprivation and some aspect of the experiences of asylum seekers and beneficiaries of international protection in Italy), focusing on differences by gender and nationality. A discussion follows, as well as conclusions providing some insights on the complex Italian legal and operational framework.

### Sizing and understanding the phenomenon in Italy within the European context

According to the Dublin III Regulation (Reg. No. 604/2013), asylum seekers are obliged to submit their applications in the first EU country they entered. This places an unequal burden on different countries and often compels individuals to stay in states they have not chosen and where they may lack support networks. This transient status intersects with procedural hurdles, leaving them in an extended limbo and undermining efforts at community settlement.

In 2024, the EU adopted the New Pact on Migration and Asylum, which seeks to promote greater solidarity and fairer responsibility-sharing among Member States, although its measures will not come into effect until 2026. While the New Pact seeks to create a more coordinated and efficient asylum system within the EU, its implementation has raised concerns about unequal treatment among asylum seekers [21]. For instance, the differential reception of Ukrainian refugees – granted swift protection under the EU Temporary Protection Directive – compared to asylum seekers from countries such as Afghanistan, Eritrea or Sudan, highlights how political priorities and racialised narratives influence access to protection. The debate on deserving and undeserving refugees is particularly underscored in the last years, also in Italy [22].

In 2024, the European Union saw a notable shift in asylum dynamics. First-time applications for international protection dropped by just over 13% from the previous year, falling to approximately 912,000 requests [23]. This decline marked a general slowdown across the continent, though the picture varied considerably between member states.

Italy emerged as a major recipient country, receiving over 151,000 first-time applications and accounting for nearly one-sixth of the EU total – a figure that represented a 15.7% increase from 2023. Only Germany and Spain received more applications, whilst France and Greece followed closely behind. These five countries collectively absorbed more than 80% of all asylum requests submitted across the EU [23].

First-time applicants made up the overwhelming majority (over 91%) of all asylum seekers in the EU, with repeat applicants – those reapplying after previous rejections – numbering just over 83,000, a modest increase from the year before. Italy also featured prominently in accelerated procedures for cases deemed unlikely to succeed, processing nearly 25,000 applications through this fast-track system, second only to France [24,25].

These figures about accelerated asylum procedure may suggest an effort to improve administrative efficiency but they also reflect a more restrictive approach under the Meloni government, which has been in office since 2022. As noted in the ECRE-AIDA reports [24,25], the expansion of accelerated and border procedures has raised concerns about the erosion of asylum seekers’ rights, particularly regarding adequate preparation time and access to legal aid. Moreover, the broader national overview highlights that the increasing reliance on such procedures may not always comply with EU standards, especially when applied to vulnerable individuals or those from countries designated as “safe”.

The nationality profile of asylum seekers in Italy diverges markedly from the EU pattern. Across the Union, Syrian citizens remained by far the largest group, followed by Venezuelans, Afghans, Colombians and Turkish nationals. Asilum applications in Italy, however, reflected different migration dynamics and routes. Bangladeshi nationals formed the largest cohort with nearly 33,000 applications, followed by Peruvians and Pakistanis. Syrian applicants, whilst dominant elsewhere, ranked only fourth in Italy, with Afghans in fifth place. This distinct pattern reflects Italy’s position on Mediterranean routes and suggests a migration flow increasingly oriented towards Southern Asia rather than the Middle East.

By the end of 2024, over 1.24 million asylum applications remained pending across the EU, representing an 8.5% increase from 2023. Italy’s backlog exceeded 207,000 cases, placing it third behind Germany and Spain in terms of unresolved applications.

This situation is not merely the result of increased demand; rather, it reflects chronic underinvestment in the personnel and infrastructure needed to process applications efficiently [24] and administrative barriers, such as the requirement of unnecessary documentation like a passport or a formal address.

Beyond these issues, the outcomes of asylum decisions reveal important trends in protection recognition. In 2024, roughly 388,000 asylum seekers received positive decisions at first instance across the EU, split fairly evenly between refugee status, subsidiary protection and humanitarian status [2]. The overall recognition rate stood at just above 51%.

Italy’s recognition rate stood at 36%, meaning nearly two-thirds of applications were rejected. Among those granted protection, the distribution was relatively balanced: humanitarian status proved slightly more common than subsidiary protection, with refugee status granted in just under 8% of cases. This pattern continued a trend observed since 2023, when the overall recognition rate had already declined from 47% in 2022 to 37% [24]. The shift towards granting subsidiary and national forms of protection, rather than refugee status, may be indicative of evolving policy approaches and the challenges faced by the asylum system in adapting to increasing application numbers and complex migration dynamics.

The growing reliance on granting subsidiary and national forms of protection – rather than full refugee status – may reflect both a shift in policy orientation and the limitations of the current asylum system in addressing the increasing volume and complexity of protection claims. This trend suggests that governments are adapting their responses to evolving migration realities, often balancing legal obligations with political and administrative pressures, while also navigating the blurred lines between forced displacement and broader forms of migration.

## Materials and methods

### The survey

#### Eligibility criteria.

The *ItRAS* survey targeted adults (18 years and above) who had been granted various forms of international protection and individuals with a previous asylum-seeking history.

This study explicitly distinguishes between different legal statuses within protection-related migration, as these categories entail substantially different rights, degrees of legal stability and access to reception and integration measures, particularly in the Italian context.

In Italy, asylum seekers are individuals who have applied for international protection and hold a temporary residence permit while their application is under examination; during this phase, access to employment, welfare benefits and housing is conditional and often limited, and individuals are primarily hosted within the asylum reception system. Beneficiaries of international protection – including recognised refugees and holders of subsidiary protection – are granted a more stable legal status, longer residence permits and broader access to the labour market, social services and family reunification.

The study also considers beneficiaries of temporary protection, a legal framework activated at the EU level following the Russian invasion of Ukraine in 2022. In the Italian context, this status is characterised by immediate access to residence permits, employment, healthcare and dedicated reception arrangements that differ substantially from those available to asylum seekers and other protection holders. Finally, the analysis includes individuals granted national forms of protection under Italian law, commonly referred to as “humanitarian” or “special” protection, which provide intermediate and often more precarious forms of legal stability, with rights and duration that have varied across policy reforms and over time.

Importantly, the survey also includes individuals who, after having initially been granted a residence permit for protection reasons, subsequently changed their legal status. This group comprises respondents who converted their permit into a non–protection-related residence permit (e.g., for employment or family reasons), as well as those who transitioned to more stable statuses, such as the EU long-term residence permit or Italian citizenship. Although these individuals no longer hold a formal protection status at the time of the interview, they share a refugee background and experienced the asylum and protection system upon arrival. They are also among the most difficult populations to capture in surveys focused on forced migration, yet they are crucial for understanding longer-term trajectories of settlement and integration among individuals with a refugee background.

The study only included adult individuals who arrived in Italy no earlier than 2011 and who had resided in the country for at least six months prior to the date of their interview. This temporal restriction was applied in order to focus the analysis on migrants with a refugee background who arrived in Italy within the context of the most significant and structurally distinct refugee movements affecting Europe in the last decade. Specifically, the year 2011 marks a critical turning point in contemporary forced migration to Europe and Italy, coinciding with the onset of the Arab Spring and the subsequent increase in mixed migration and asylum flows across the Central Mediterranean route. From this period onwards, Italy experienced a sustained rise in asylum applications, changes in the composition of countries of origin and substantial transformations in both European and national asylum and reception policies. By excluding individuals who arrived before 2011, the study ensures greater comparability in terms of migration trajectories, legal frameworks, reception conditions and integration opportunities experienced by respondents. This choice reduces heterogeneity linked to markedly different pre-2011 asylum regimes and socio-political contexts, thereby strengthening the internal coherence of the analytical sample and the interpretability of the results.

#### Ethical compliance and bioethical approval.

The study protocol, including the sampling design, survey instrument and consent procedure, was reviewed and approved by the Bioethics Committee of the University of Palermo (Opinion No. 212/2024, issued on April 24, 2024). All respondents were at least 18 years old and provided informed consent prior to participation. At first contact, interviewers presented participants with a digital informed consent form on their tablet screen. Respondents were required to read and explicitly agree to the consent form before proceeding with the questionnaire. No interview was conducted without prior consent.

The consent form clarified the voluntary nature of participation, the right to withdraw at any point or skip questions and the complete anonymity of data collection. It was clearly stated that exercising any of these options would have no consequences for the participants. No personal identifiers (such as names, addresses, or ID numbers) were collected or stored. Data were recorded and processed in an anonymous and aggregate form.

#### The questionnaire.

The *ItRAS* is based on an *ad hoc* questionnaire, aligning with one of the primary objectives of the AVRAI project: measuring the vulnerability of AS&Rs in Italy. The questionnaire was designed to be as concise as possible while ensuring comparability with datasets collected on the same target population in other European countries, that is, with European refugee surveys such as the German IAB-BAMF-SOEP Refugee Survey and the Austrian ReHIS. Its development followed several key steps. First, a literature review was conducted and the theoretical framework was defined to identify the relevant dimensions to be investigated. A focus group with experts and professionals in the field was then organised to validate the questionnaire’s basic structure by analysing the perspectives that emerged during the discussion. This aimed to identify eventual specificities related to vulnerabilities in high-income countries such as Italy.

The next phase involved formulating the survey questions. This task was developed through a consensus process inside the national research group, composed of ten academic experts in the field from the Universities of Palermo, Bologna, Milano, Milano Bicocca and the University of Calabria. Whenever possible, previously validated question formulations, from other survey questionnaires, were adopted to allow further comparisons. Some items were drawn from the Italian special module for foreign nationals in the European Union Statistics on Income and Living Conditions (EU-SILC, see [26]) and from the FAO [27]. When alignment with existing surveys was not feasible, new *ad hoc* questions were developed and tested.

In its final version, the questionnaire consists of seven sections, each examining different aspects of AS&Rs’ living conditions. Additionally, there is a section dedicated to assessing respondents’ eligibility based on the selection criteria previously introduced (legal status, age, and year of arrival in Italy).

**Fig 1:**
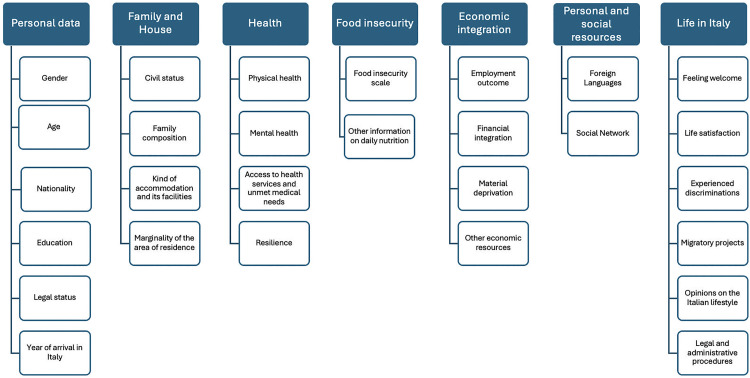
Questionnaire structure.

The various sections explore key dimensions relevant to assessing AS&Rs’ vulnerability, including personal characteristics, family and housing conditions, health, food insecurity, economic integration, personal and social resources and broader aspects of life in Italy (see Fig 1).

Given the nature of the research problem, the questionnaire investigates objective and subjective dimensions. Objective aspects include the presence or absence of specific conditions or the degree to which they manifest, while subjective dimensions relate to respondents’ perceptions, attitudes and opinions. In this case, responses were measured using Likert scales.

#### The centre sampling technique.

We drew the sample for this study using the Centre Sampling Technique (CST), a method employed to survey elusive populations. Baio et al. [28] developed CST to study migrant communities in Lombardy (a northern Italian region), but, more in general, this framework is suitable to obtain a semi-probability sampling even when complete sampling frames are unavailable, inaccessible, or unreliable and has been applied to a variety of contexts [29–31]. This method relies on the assumption that individuals belonging to a hard-to-reach population frequently attend a set of identifiable public spaces, which may include places of worship, specific shops, centres for aggregation, associations and so on. These spaces, referred to as “centres,” serve as observable proxies for an elusive population and form the basis of the CST sampling universe.

The application of CST follows a two-stage sampling design. In the first stage, researchers construct a sampling frame including all the relevant centres where the population of interest is more likely to attend. This phase involves identifying a comprehensive list of centres likely to be attended by the target population, ensuring diversity in the types of services offered, geographic distribution, and the demographic composition of their users. In our case, we identified 13 categories of places refugees and asylum seekers could spend time in, such as centres providing services to migrants and refugees, official reception centres, places of worship, churches and temples, food and general markets and malls.

The second stage consists of selecting individuals to take part in the survey within each of these centres. This must be done by enumerators directly on site. Once individuals agree to take part in the survey, a specific section of the questionnaire collects data on individuals’ centre-attendance patterns. As is explained in the following, this information is used to assess the probability of a certain individual taking part in the survey. This step is fundamental to computing *ex-post* sampling weights and ensuring representativeness of the sample. In fact, respondents may attend multiple centres, increasing their probability of being included in the sample; however, by mapping and quantifying these patterns of interaction, CST allows for meaningful inference regarding population-level parameters.

#### Fieldwork.

Interviews were conducted face-to-face using a CAPI (Computer-Assisted Personal Interviewing) interface. Interviewers were recruited by a company specialised in statistical surveys of migrants in Italy, hired by the AVRAI project. The company that hired and coordinated the fieldworkers is not involved in the research and does not have any right over the data.

To encourage participation, build trust and ensure linguistic and cultural sensitivity toward the surveyed communities, all interviewers were selected among individuals with migratory backgrounds or from communities represented in the survey. Interviewer training began in late January and was primarily conducted in person, with online sessions offered when in-person training was not feasible. Members of the AVRAI research team illustrated the aims of the survey and drew attention to the process of collecting data according to ethical principles in scientific research and considerations regarding anonymity, privacy and informed consent. Training also covered explanations of the questionnaire and its structure and content and usage of survey software.

Interviews were conducted in one of the questionnaire’s available languages (Italian, English, French and Arabic) after the consent form was explained in their preferred language. When respondents could not understand any of these languages, cultural mediators provided support.

#### The sample.

The *ItRAS* was coordinated by the University of Palermo, in partnership with the Universities of Bologna, Milan, Milan “Bicocca” and the University of Calabria. Due to time and budget constraints, the survey was carried out in the period from the 30^th^ of April to the 31^st^ of July 2024, only across nine Italian regions, encompassing the country’s three macro-regions: the South and major islands, Central Italy, and Northern Italy. Sixty-six municipalities of varying sizes – small, medium and large – were randomly selected for participation. They are all municipalities located within provinces that host a Territorial Commission (the body responsible for decisions on first-instance asylum applications).

The rejection rate among eligible survey participants was 45%. Lower rejection rates occurred in reception centres and facilities offering services to foreign nationals, where local staff facilitated participation. We observed higher rejection rates in areas where individuals typically had less time available, such as markets, shops, malls and workplaces. The final sample comprised 1,327 individuals. Among them, 75% held some form of protection, including 18% with refugee status and 224 Ukrainian nationals with temporary protection. Asylum seekers constituted the remaining 25% of the sample. The most represented nationalities were Ukrainian (17%), Pakistani (9.6%) and Bangladeshi (9.2%). Approximately half of the respondents were citizens of African countries, followed by Asian (27%), European (18%), South American (5%) and stateless individuals (2%). The largest proportion of the sample (40%) resided in Northern Italy, followed by residents of Central Italy (34%) and Southern Italy (25%).

#### Computation of sampling weights.

To allow statistical inference from data collected with the *ItRAS* survey on AS&Rs and to control for the distortion introduced during the execution of the survey, a set of weights was developed to allow for generalising sample results to the whole Italian target population.

Calculation of national weights is initially based on the information on the kind of centres attended by the interviewees. For each statistical unit included in the sample (i.e., for each AS&R), it was possible (at the time of the interview) to record the typology of centres where contact was established and the respondent selected and those others usually attended by the interviewee. This information is arranged in a vector which corresponds to an individual’s “affiliation profile”. Hence, it is possible to determine (retrospectively) the individual inclusion probabilities for each sampled unit assuming that: a) the higher the mobility (accounted trough the number of centres attended) of AS&Rs in the municipal territory, the higher their probability of being part of the sample; b) the higher the number of respondents associated with each centre (“attendance frequencies”), the lower the probability for the attender to be selected as respondent of the survey.

Consequently, a set of weighting coefficients can be developed, based on this profile, to eliminate distortion. The use of weights ensures that the (weighted) sample has, in terms of the distribution of affiliation profiles with the centres of the N sampled units, the same representativeness characteristics as a hypothetical simple random sample obtained through standard procedures that guarantee statistical representativeness.

The second step leaned on external information obtained from official data on residence permits, from the Italian National Statistical Institute (ISTAT), to allow for a correction of eventual disproportion in gender and citizenship. Correction coefficients are derived for each gender-nationality group and applied to the base weights to yield the “final adjusted weights” more accurately representing the target population structure. The ISTAT certified, as of January 1st, 2023, 350,345 holders of international protection permits in Italy. Expansion factors were also computed for the representativeness of North, Centre and South of Italy. Unfortunately, the sample is not representative by legal status due to its small sample size.

## Results

The following sections provide some descriptive results of the main investigated areas of the *ItRAS* questionnaire, covering only a part of those introduced in Fig 1. All the analyses (including graphs) refer to weighted data, so that results can be assumed as a generalisation to the whole national target population. Analyses are presented by nationality and gender, with some additional in-depth considerations.

For the sake of conciseness, countries of origin of AS&Rs were grouped into 10 geographic areas of interest in migration studies. In particular, four groups correspond to a single nationality – *Bangladesh, Nigeria, Pakistan* and *Ukraine*. These countries represent analytically distinct and policy-relevant cases within the Italian asylum framework, reflecting different combinations of recognition rates, legal pathways and institutional responses. Bangladesh and Pakistan are characterised by low asylum recognition rates but a substantial presence of individuals who transition from asylum seeking to labour- or family-based legal statuses. Nigeria represents a case of heterogeneous protection outcomes and complex vulnerability profiles, while Ukraine constitutes a unique case of forced displacement managed primarily through the EU temporary protection regime. These groups are also the most represented in our sample, each with more than 100 cases. Then we have six groups of countries sharing some border or economic and political ties, such as *Horn of Africa* (Eritrea, Ethiopia, Somalia), *Middle East and North Africa* – i.e., the MENA region– (including Algeria, Egypt, Iran, Iraq, Lebanon, Libya, Morocco, Mauritania, Occupied Palestinian Territories, Syria, Tunisia, Turkey), *Other Africa* (including Angola, Benin, Botswana, Burkina Faso, Cameroon, Central African Republic, Chad, Congo, Congo Republic Dem., Ivory Coast, Gabon, Gambia, Ghana, Guinea, Liberia, Malawi, Mali, Niger, Senegal, Sierra Leone, Sudan, Togo, Togo, Uganda, Zambia), the *South-East Asia* (including Afghanistan, China, Philippines, India, Kazakhstan, Nepal, Sri Lanka, Tajikistan), *Latin America* (Brazil, Colombia, Ecuador, El Salvador, Jamaica, Mexico, Pakistan, Venezuela), *Other EU* (includes the few stateless interviewed and the following European countries: Albania, Moldova, Romania, Russia, Slovenia).

### Legal dimension and performance in Italy

The legal dimension plays a crucial role in this study, as it determines the eligibility of selected individuals. A first exploration of our data (Table 1) reveals that the majority of sampled individuals, approximately 69.1%, currently hold a residence permit for protection. Asylum seekers represent 17.9% of the sample, while 8.2% have obtained an EU residence permit for long-term residents or Italian citizenship. Additionally, 4.8% have transitioned to a residence permit for non-protection reasons.

**Table 1:** Detailed legal status by gender of *ItRAS* interviewees (weighted data).

Legal Status	Sex				Sex		
	M	F	Total		M	F	Total
Protection-related permits	57.6	81.9	69.1	• Refugee status	16.9	9.7	13.5
				• Subsidiary protection	12.2	8.1	10.2
				• Humanitarian/special protection	14.1	6.4	10.5
				• Temporary protection	14.5	57.7	34.8
Asylum seekers	26.1	8.7	17.9	• Appeal after asylum request rejection	6.1	1.1	3.7
				• Asylum seeker waiting for the first decision	19.0	7.2	13.4
				• Asylum seeker waiting for the first decision on asylum and on the 2020 amnesty application	1.1	0.5	0.8
Other fixed-term permitsnon-asylum related	6.9	2.4	4.8	• Valid	5.7	2.0	4.0
				• Undergoing renovation	1.2	0.3	0.8
Unlimited term legal status	9.4	7.0	8.2	• Italian citizenship	0.6	1.2	0.8
				• EU residence permit for long-term residents	8.8	5.8	7.4
Total	100.0	100.0	100.0		100.0	100.0	100.0

Gender differences emerge in the data, with women being overrepresented among those holding protection permits, while men account for a higher proportion of asylum seekers. Men are also more likely to hold fixed-term permits for other reasons and to transition to citizenship or long-term resident status. The significant presence of Ukrainian migrants, who make up 65% of the women in the sample, largely explains this gendered distribution, as 82.2% of them hold temporary protection status.

Legal status also varies significantly depending on individuals’ regions of origin (Table 2). Asylum seekers are most prevalent among those from the Middle East and North Africa (MENA), Bangladesh, and Pakistan. Protection-related permits are more common among Ukrainians, Nigerians, other European nationals and Latin Americans, while individuals from the Horn of Africa and Europe more frequently hold long-term residence permits or citizenship.

**Table 2:** Legal status by geographical area of origin of *ItRAS* interviewees (weighted data).

	Bangladesh	Nigeria	Pakistan	Ukraine	Horn of Africa	MENA	Other Africa	Other South-East Asia	Latin America	Other Europe & Stateless	Total
Protection-related permits	23.7	68.5	38.0	94.2	69.3	32.3	58.6	46.3	63.6	74.4	69.1
Asylum Seeker	46.0	14.9	48.9	0.3	11.9	57.5	22.5	34.2	15.2	2.3	17.9
Unlimited term legal status	9.5	7.8	9.2	4.9	16.8	7.0	11.1	15.0	10.4	17.1	8.2
Other fixed-term legal status	20.9	8.9	4.0	0.6	2.0	3.3	7.8	4.5	10.8	6.2	4.8
Total	100.0	100.0	100.0	100.0	100.0	100.0	100.0	100.0	100.0	100.0	100.0

These findings underscore diversity of legal trajectories among *ItRAS* interviewees, which vary by country of origin, gender and migration history. The observed distribution closely mirrors recent patterns in humanitarian inflows to Italy (i.e., those incoming flows of people asking for international protection). In the *ItRAS* data, individuals from African countries – who were overrepresented among sea arrivals to Italy prior to the COVID-19 pandemic [32] – are more likely to hold protection-related permits. By contrast, interviewees from Bangladesh, Pakistan, and the MENA region – groups more prevalent among post-pandemic arrivals to Italy [32] – show the highest proportions of individuals whose asylum applications are still pending. Finally, most Ukrainian interviewees hold temporary protection permits, as this status is granted without requiring an individual assessment of the asylum claim.

Long waiting times for status recognition are acknowledged as a driver of uncertainty and vulnerability. Among the 947 respondents who had received a first decision on their asylum application, the average waiting time stood at 10.2 months, with a median of 6.5 months – highlighting a wide disparity in individual experiences. For the 214 individuals still awaiting a decision, the average waiting time is slightly higher at 10.3 months, with a median of 7.5 months, suggesting that prolonged uncertainty remains a key challenge for many.

Noticeably, given that by design we selected only people who arrived in Italy since 2011, the average time since arrival among respondents is 4.5 years. Ukrainians, in particular, have resided in Italy for an average of 2.9 years, indicating that most arrived shortly before or after the onset of the 2022 war in their country. Among the other most represented nationalities in our sample, Pakistanis had been in Italy for an average of 4.8 years, while Nigerians reported an average of 6.2 years.

### Health

Established empirical evidence suggests that migrants often arrive in destination countries in better health than both native populations and their co-nationals remaining in the country of origin. This phenomenon, often referred to as the “healthy migrant effect”, is typically observed among “economic migrants” (those who voluntarily, hence not forcibly, migrate seeking better economic opportunities), who represent a positively selected group in terms of health and resilience [33,34]. However, this pattern does not hold for asylum seekers and refugees, who constitute a distinct and far less self-selected migrant population. Compared to adult economic migrants, indeed, they are less positively selected and more likely to have experienced traumatic and psychologically distressing pre-migration, migration, and post-migration events, which can severely affect their mental and physical health over time [35–37]. Despite this, the mental and physical health of AS&R in high- and middle-income countries remains under-researched [38] and the Italian context is no exception [18,39].

#### Self-reported health.

**Fig 2:**
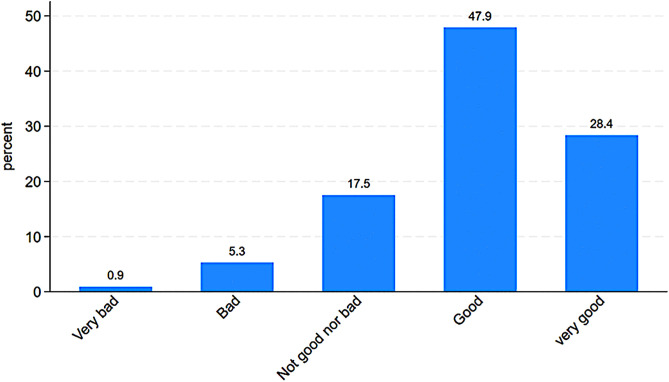
Percentage distribution of self-reported health.

Unpacking the first trends in the *ItRAS* data, we see that overall, 77.3% of AS&Rs report good or very good self-rated health (SRH), with 28.3% specifically reporting very good health and 6.2% reporting poor or very poor health (Fig 2). In comparison, in 2022, 68.7% of Italians reported good or very good SRH (our elaboration on [40]), a difference that may be only partly attributable to different age structures. However, similar age-group comparisons reveal more nuance: while young interviewees report worse SRH than their Italian peers, adults AS&Rs tend to report better SRH than Italian adults.

Country/area of origin also matters: *ItRAS* interviewees from Pakistan and sub-Saharan Africa tend to report better SRH, while those from Bangladesh, Nigeria, the Horn of Africa, the MENA region, and Ukraine report higher levels of poor or very poor SRH. Interestingly, the year of arrival seems to affect perceived health. Those who arrived in Italy between 2011 and 2014 report the best SRH, while those who arrived between 2015 and 2018 report the worst. The effect of length of stay is non-linear and may reflect shifts in the composition of incoming flows by country of origin.

Gender differences are also evident: 33.4% of men report very good health compared to 22.4% of women, whereas women are more likely to report merely good health (52.1% vs. 44.5%). During their stay in Italy, most *ItRAS*’s refugees and asylum seekers report stable health conditions. However, 17.5% note an improvement, while 19.7% report deterioration. Women are more likely to report no change in their SRH over time.

Unmet health needs affect 14.3% of the sample (see Table 3), compared to only 6% of Italians (ISTAT). Women report higher levels of unmet needs than men (15.9% vs. 12.9%). Higher levels are also observed among individuals from the MENA region (21.6%) and sub-Saharan Africa (16.4%), whereas individuals from Bangladesh report the lowest levels. AS&Rs with longer stays are more likely to report unmet needs (17.4%). Among individuals who reported needing care, 22.1% did not receive it. The most recent arrivals and those with the longest stays report slightly higher levels of unmet needs (24.7% and 23.7%, respectively).

**Table 3:** Very good health and mental health and unmet needs among AS&Rs.

Health measure	Percentage
Very good self-rated health (SRH)	28.3%
Very good mental health (MH)	20.9%
Unmet health needs	14.3%
Unmet health needs among those needing a visit or treatment	22.1%

#### Mental health.

A “very good” mental health, defined as almost always feeling happy and calm in the past four weeks, is reported by 20.9% of the sample (see Table 3), a lower rate than for very good SRH (28.3%). The mental health dimension was assessed using two questions from the AVRAI survey, following the model by Ware et al. [41]. Respondents reported how frequently they felt sad and how often they felt calm during the previous four weeks. The sadness dimension was reversed and individuals scoring “almost always” or “always” on both items were classified as having very good mental health. In this dimension, women tend to report poorer mental health. AS&Rs from Nigeria and the Horn of Africa have the worst scores, while those from the MENA region, sub-Saharan Africa and Ukraine fare better. Mental health issues are more prevalent among recent arrivals but tend to improve with length of stay.

These findings confirm that while asylum seekers and beneficiaries of international protection often report relatively good self-rated health, especially when compared to national averages, there are important disparities across gender, origin and time of arrival. Women consistently report worse outcomes in both physical and mental health dimensions, and unmet health needs remain a significant issue, particularly among those with longer stays or recent arrivals. Mental health emerges as a key area of concern, with only a minority reporting very good well-being, highlighting the psychological toll of migration and forced displacement.

**Fig 3:**
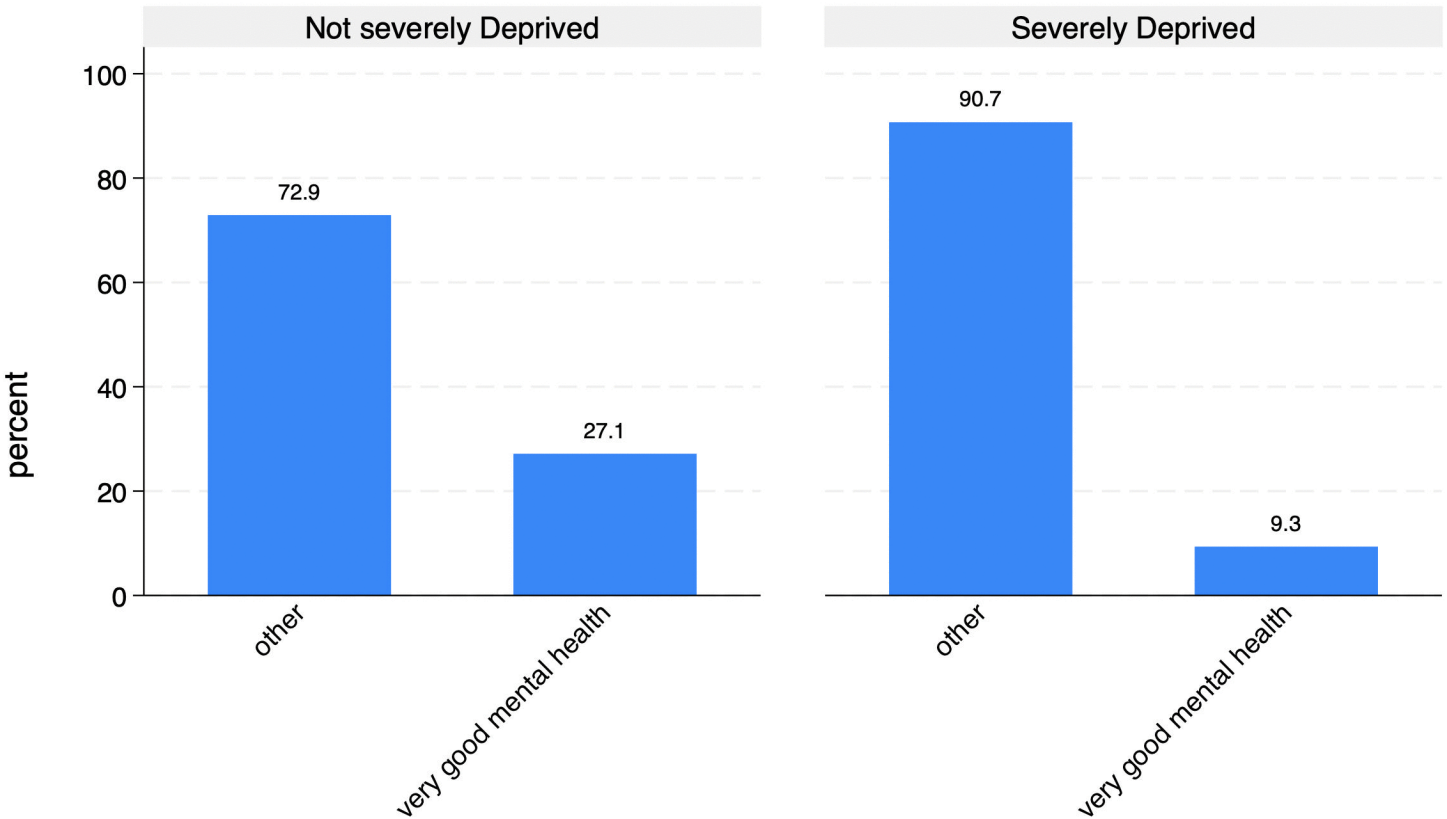
Distribution of mental health by level of material deprivation (%).

Having a partner – especially in a cohabiting relationship- has a protective effect on mental health, confirming the importance of both emotional and practical support within the couple [42]. In addition, severe material deprivation is strongly associated with poor mental health: 27.1% of our target population in not-severe material deprivation have very good mental health compared to 9.3% of those materially deprived (Fig 3).

These results underscore the need for health policies and services tailored to the specific vulnerabilities of AS&R populations. A combination of structural and individual-level factors likely shapes these outcomes. Legal status uncertainty, barriers to healthcare access and experiences of discrimination can negatively affect both physical and mental health. At the same time, gendered migration trajectories and country of origin-specific vulnerabilities contribute to the heterogeneity in health outcomes observed across groups.

### Resilience

Resilience is increasingly recognised as a key protective factor for the psychosocial health of forced migrants, highlighting the need for a shift from treatment-focused approaches to those that emphasise prevention and resilience promotion [43].

Psychologists have long engaged in an extensive debate over the conceptualisation and measurement of resilience. The American Psychological Association [44] defines resilience as “the process and outcome of successfully adapting to difficult or challenging life experiences, especially through mental, emotional, and behavioural flexibility and adjustment to external and internal demands”. In this sense, resilience refers to the individual’s capacity to cope with and rebound from adversity and external challenges. As a personal trait, it can be seen as an individual’s adaptability and strength (see [45], as cited in [46]), but also an ability that exists “in relation to something,” such as adversity or external challenges [47].

The *ItRAS* contributes to the resilience literature by collecting quantitative data in the Italian context, introducing in the questionnaire a battery of items drawn from some resilience scales. Here, we focus specifically on resilience as a personal trait, emphasising individual adaptability and strength. These data refer to the answers to the question *“I am able to adapt when changes occur”* measured on a 5-point Likert scale, ranging from 1 (Not at all true) to 5 (Almost always true), allowing for capturing variations in respondents’ perceptions.

**Fig 4:**
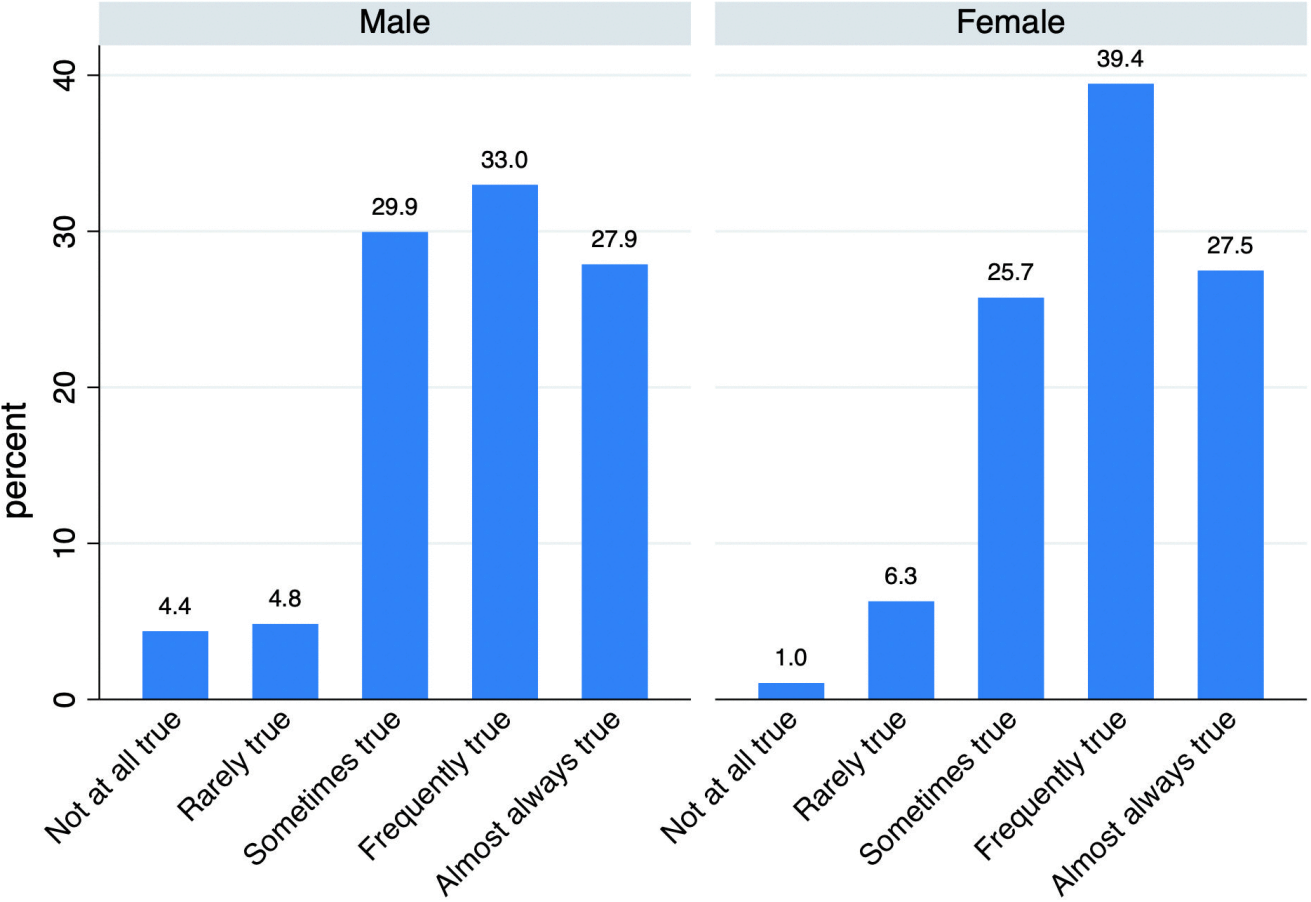
Resilience by gender (%).

The survey results reveal that most respondents declare a moderate to high level of resilience (Fig 4). Regarding the ability to adapt to changes, 60.9% of men reported “frequently true” or “almost always true,” compared with 66.9% among women. Less than 10% of men answer “Not at all true” or “Rarely true” about their adapting capacity.

**Fig 5:**
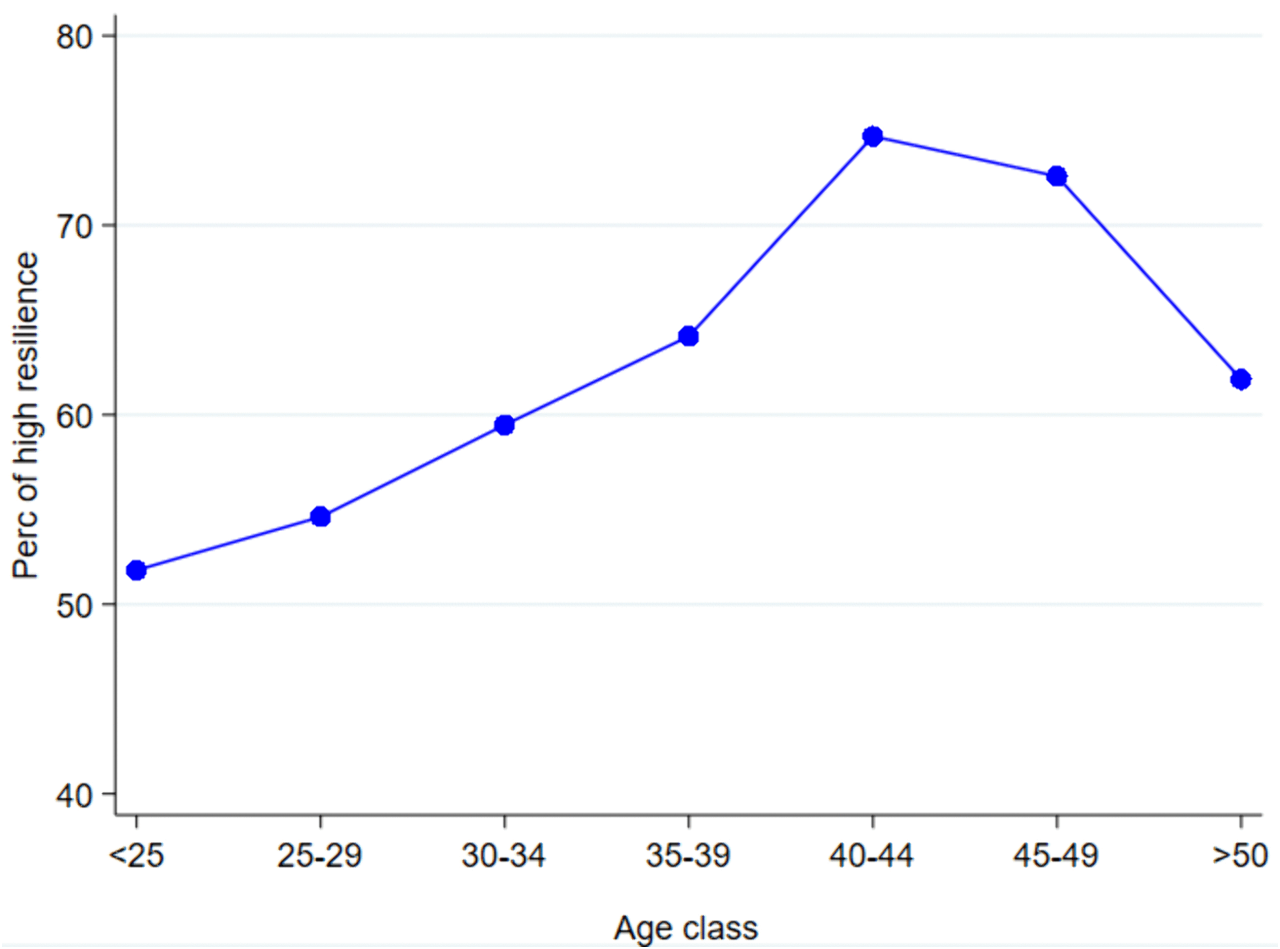
Resilience by age class (%). * The percentage of resilience aggregates those who answer frequently or almost always true.

The analysis of resilience by age (Fig 5) shows that percentage of highly resilient follows an inverted “U” shape, with the lowest incidences among younger individuals (around 50% of respondents answered “frequently” or “almost always true” among those under 25). After peaking at 75% among those aged 40–44, resilience is lower in the following age groups, reaching about 60% among those aged 50 and older.

**Fig 6:**
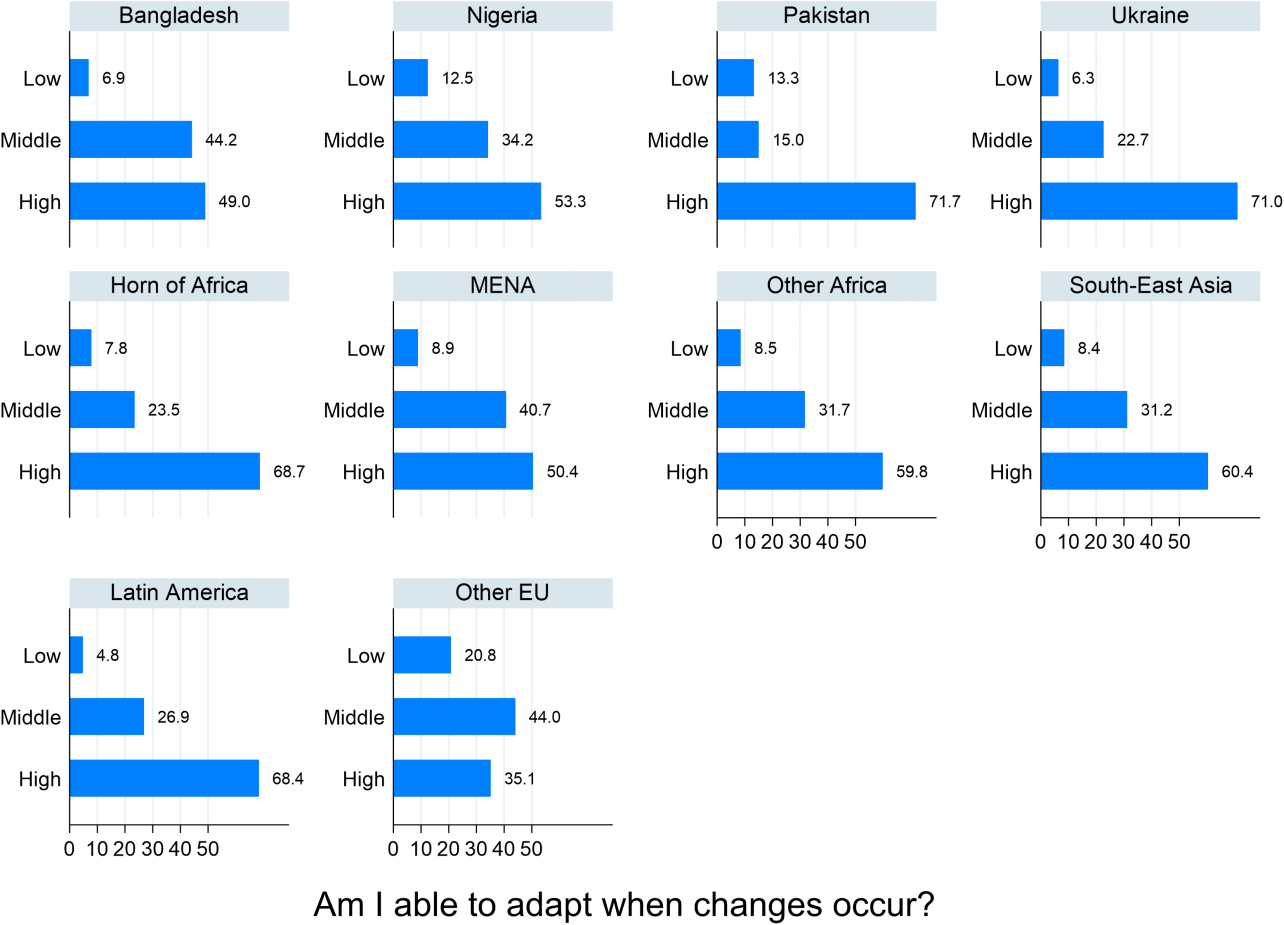
Resilience* by main nationalities and area of origin (%). * Low includes the categories “not at all true” and “Rarely true”; Middle includes “Sometimes true”; High includes “Frequently true” and “Almost always true”.

Resilience also varies significantly depending on individuals’ regions of origin. Among the oversampled communities, Ukrainians and Pakistanis present the highest levels of resilience, while Bangladesh is the least resilient community. Considering the geographical origin, those from the Horn of Africa and Latin America present the highest levels (Fig 6). The ‘Other Europeans’ category – that also includes those from EU countries – shows the lowest levels of resilience. This supports the idea that resilience is a successful adaptation process to difficulties and challenging life experiences, a trait that activates in the face of adversity.

One of the primary individual characteristics associated with resilience is education. Findings indicate that resilience as adaptation is consistently higher among individuals with higher education (here including high school degree, bachelor’s degree and above). Specifically, 66.6% of those with higher education exhibit high adaptation [95% CI: 62.4–70.5], compared to 50.0% of those with lower education [95% CI: 43.7–56.1]. A similar pattern emerges for the ability to recover quickly, with 68.4% of highly educated individuals [95% CI: 63.5–73.0] reporting it compared to 55.0% of those with lower education [95% CI: 50.3–59.5].

### Food security

Food security is defined as a situation where all people, at all times, have physical, social, and economic access to sufficient, safe, and nutritious food that meets their dietary needs and food preferences for an active and healthy life [48,49]. This concept is inherently multidimensional, extending beyond mere availability to encompass other critical dimensions, such as access to food, proper utilisation, and stability over time [50].

In high-income countries, food insecurity has been linked to various factors, including being a member of a racial or ethnic minority [51]. As such, refugees and asylum seekers are particularly vulnerable to food insecurity, as, unlike other migrant groups, they often face low socio-economic status and reside in rental or government-supported housing, further exacerbating their exposure to food insecurity risk factors [52]. In fact, access to food is a common concern among immigrants and forcibly displaced migrants from diverse backgrounds, with low income and high food prices being identified as significant barriers to obtaining desired food [53].

AS&Rs' food insecurity remains nonetheless an underexplored dimension of vulnerability. The existing literature, both qualitative and quantitative, primarily focuses on the North American context (e.g., [54]), with some studies also conducted in Australia and European countries to a lesser extent (e.g., [55]). To the best of our knowledge, no study on the food security of refugees, beneficiaries of international protection and asylum seekers has yet been conducted in the Italian context.

In the context of the *ItRAS* survey, AS&Rs’ food insecurity was assessed using two main tools, both of which focus on access to food. The first is a question related to food insecurity as an outcome of material deprivation, derived from a scale developed by the European Union Survey on Income and Living Conditions (EU-SILC) to evaluate severe material deprivation among EU Member States. The question asks whether the respondent can afford a meal containing meat, fish, or a vegetarian equivalent every second day.

The second instrument implemented to evaluate food security among the AS&Rs currently living in Italy is the Food Insecurity Experience Scale (FIES), first developed by the United Nations Food and Agriculture Organization (FAO) in 2014. The FIES is an experience-based metric that measures the severity of food insecurity, relying on individuals’ direct responses to questions regarding their experiences with limited access to food due to resource constraints. It measures the severity of food insecurity, conceptualised and modelled as a latent trait, and broadly understood as the condition of not being able to freely access the food necessary for a healthy, active, and dignified life [56]. The measure is based on conditions and behaviours reported through an 8-item questionnaire, the Food Insecurity Experience Scale Survey Module (FIES-SM), which reflects the inability to access food due to lack of money or other resources.

**Fig 7:**
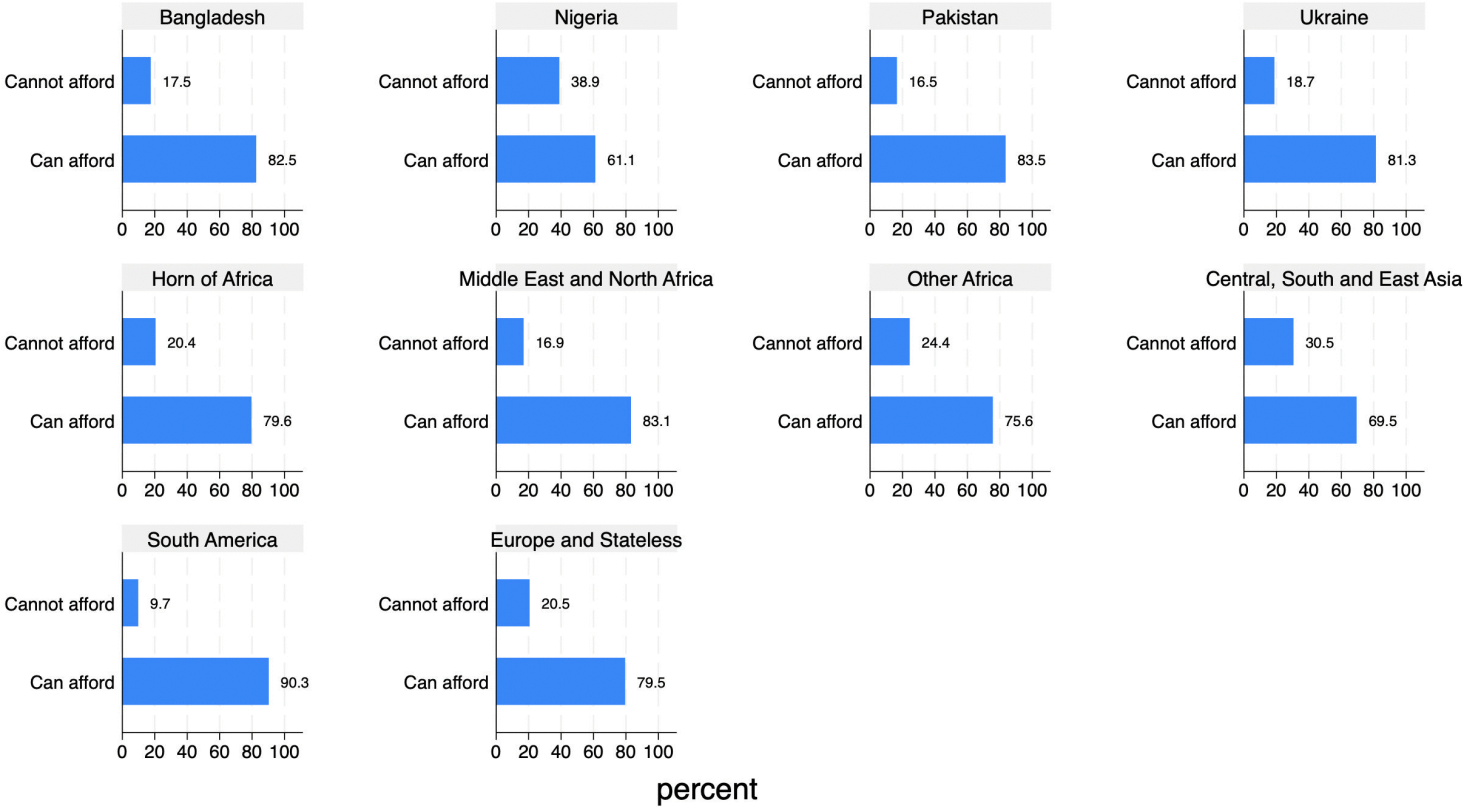
Ability to afford to eat meat or a vegetarian equivalent every second day, by geographical origin (%).

Among AS&Rs living in Italy, approximately one in five individuals (21.4%) reports being unable to afford a meal containing meat, fish, or a vegetarian equivalent every second day (see Fig 7 for the distribution by AS&Rs’ geographical origin). Nigerian AS&Rs have the highest proportion of food-insecure individuals (38.9%), followed by those from Central, South and East Asia (30.5%). For comparison, in Italy, the percentage of people unable to afford a meal with meat, fish, or a vegetarian equivalent every second day was 8.5% in 2023, while the average for the EU-27 was 9.5% [57]. The group with the closest proportion to the general Italian population is that from South America (9.7%).

Concerning the FIES, following FAO guidelines [27], we have identified four levels of food insecurity severity based on the total number of food insecurity experiences reported by respondents during the interview: 0 items indicate food security; 1–3 items correspond to mild food insecurity, 4–6 items correspond to moderate food insecurity and 7–8 items indicate severe food insecurity.

**Fig 8:**
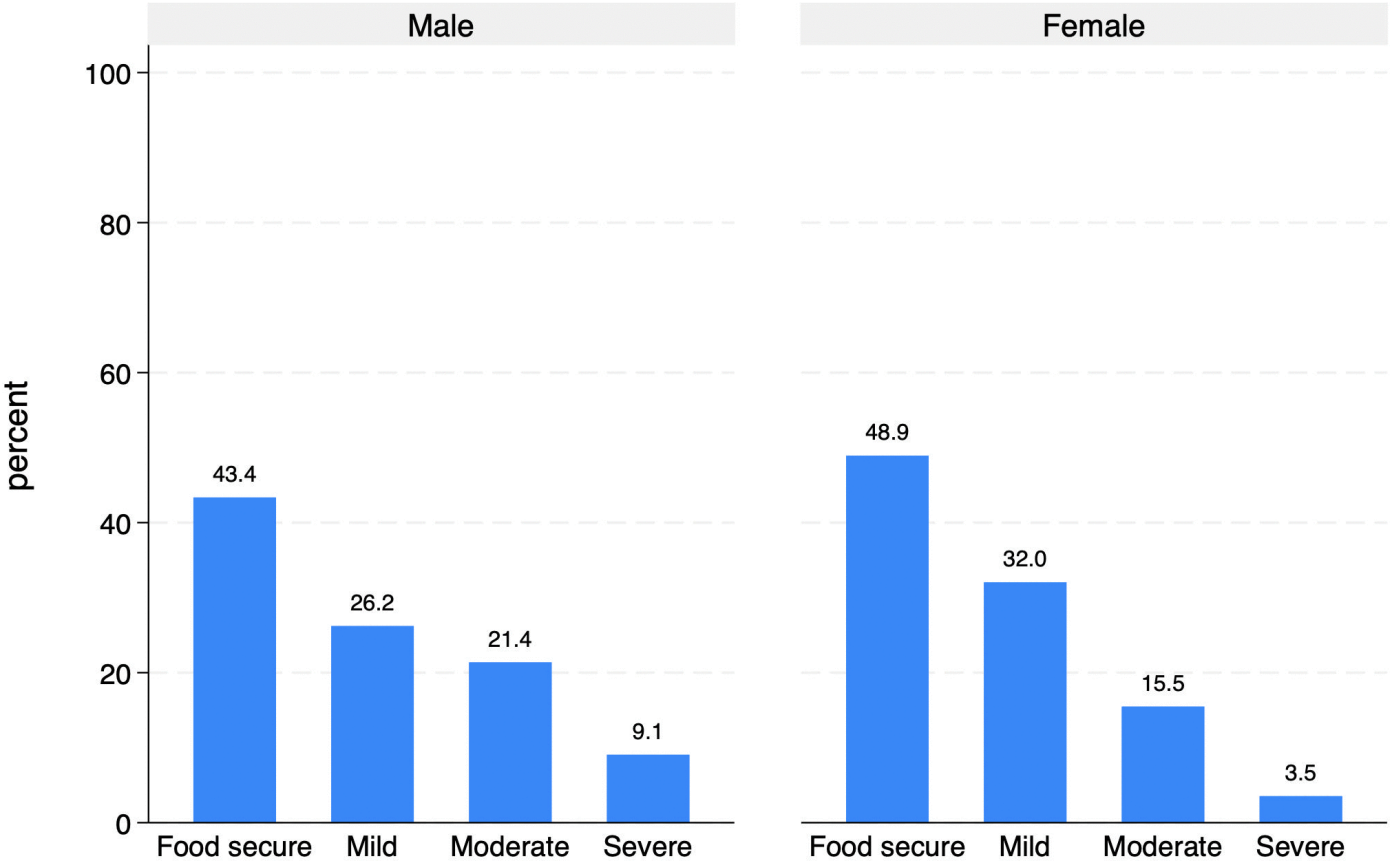
Degree of food insecurity by sex (%).

Fig 8 shows the distribution of food insecurity levels across male and female AS&Rs, suggesting a potential gender gap, with men being more represented than women in all food insecurity categories: 26.2% vs. 32.0% in the mild food insecurity group; 21.4% vs. 15.5% in the moderate food insecurity group; and 9.1% vs. 3.5% in the severe food insecurity group.

**Fig 9:**
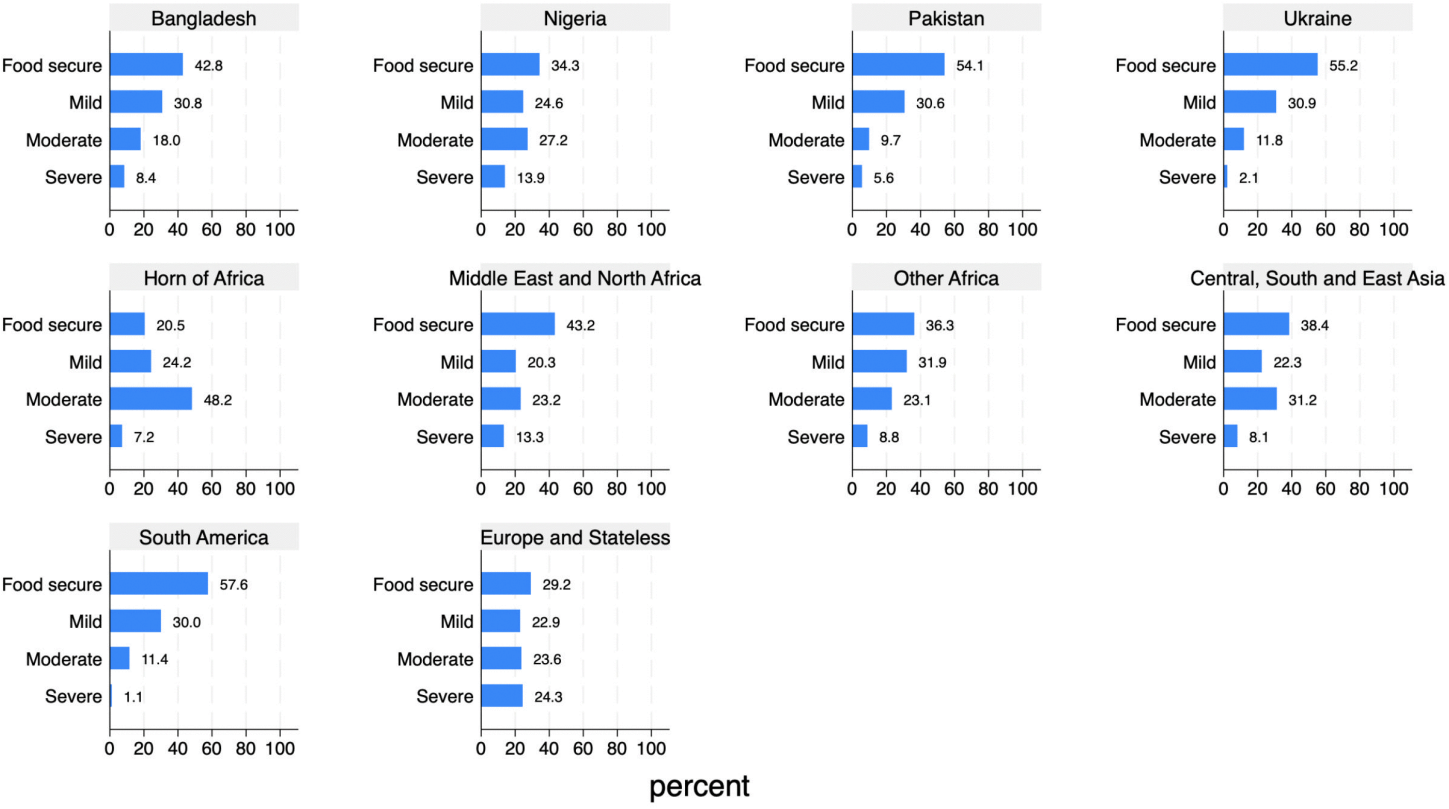
Degree of food insecurity by geographical origin (%).

Fig 9 presents the distribution of refugees, beneficiaries of international protection and asylum seekers across food insecurity levels by national or regional background. The differences observed highlight that the place of origin plays a significant role in the degree of food insecurity experienced while living as a refugee or asylum seeker in Italy. *ItRAS* interviewees from Ukraine and Latin American countries appear to experience lower levels of food insecurity compared to those from other origins. In contrast, interviewed individuals from Europe and the Stateless, Nigeria and the MENA area exhibit considerably higher levels of severe food insecurity (24.3%, 13.9%, and 13.3%, respectively) than all other groups, including Bangladeshi (8.4%) and Pakistani nationals (5.6%).

Finally, looking at the distribution of affirmative responses to the individual food security items, the most common vulnerability associated with food access among the *ItRAS* target population is the limited variety of diet – reported by 40.7% of respondents – followed by the inability to eat healthy and nutritious food – reported by 31.0% of respondents – and being worried about not having enough food to eat – reported by 26.4% of respondents. The least common experiences are, as expected, the two most severe conditions: going without eating for a whole day, reported by 9.9% of respondents, and experiencing hunger, reported by 15.6% of respondents.

Refugee backgrounds appear to play a role here as well. 2.1% of Bangladeshi AS&Rs reported going at least once without food for a whole day in the past 12 months, whereas those from African countries other than Nigeria and MENA area reported experiencing hunger (3.9%), running out of food (6.3%), and eating less than necessary (4.6%) during the last year at higher proportions than AS&Rs and asylum seekers from other nationalities. Finally, Ukrainians reported food scarcity more frequently, both in terms of quantity (7.4% skipped a meal), variety (14.3%) and quality (11.0%) than other groups. They were also more likely to express concerns about not having enough food to eat (7.4%) compared to refugees and asylum seekers from other geographical backgrounds.

### Labour market

This section investigates the labour market outcomes of AS&Rs in Italy. Existing literature suggests that refugees and asylum seekers experience lower employment levels than other migrant groups [58–60]. This may be due to several factors. Firstly, they were not prepared for migration and their decision to migrate was not primarily driven by economic considerations. Secondly, difficulties in labour market for refugees, beneficiaries of international protection and asylum seekers can be linked to the prolonged and often distressing experiences in refugee camps or asylum accommodations [61,62], the loss or devaluation of human capital and qualifications during the asylum process [58] and the effects of regional dispersal, which can limit access to co-ethnic networks that are crucial for job searching and social integration. Furthermore, they may lack access to effective social and economic support networks that facilitate employment [63] and may face greater uncertainty regarding their legal status, especially shortly after arrival, which can have a direct negative effect on labour market outcomes [59].

The literature also highlights distinct patterns of labour market integration based on gender [64,65]. Tanay et al. [66] underline the generally lower employment rates among refugee women compared to refugee men, reflecting lower activity rates and lower levels of education among refugee women.

The Italian legislative framework stipulates that asylum seekers may access the labour market 60 days after submitting their application for international protection, provided that no rejection decision has yet been issued (see Article 22 of Legislative Decree No. 142/2015). Therefore, the ability to work is subject to the issuance of the nominative certificate, the obtainment of which may further delay the period during which employment is not permitted.

Once refugee status is granted, the individual has the right to work in Italy under the same conditions as Italian citizens. This includes access to both dependent and self-employment, registration with professional bodies, vocational training, and internships. Moreover, access to public sector employment is permitted under the same conditions as those applied to European Union citizens.

**Fig 10:**
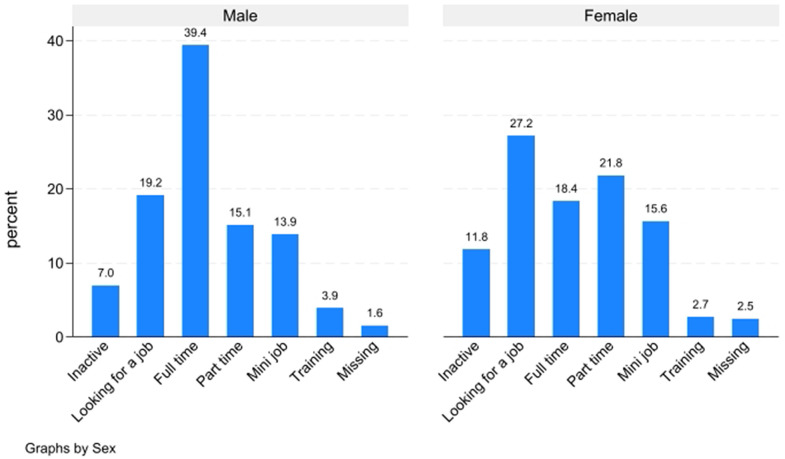
Occupational status by sex.

A first exploration of *ItRAS* data (Fig 10) reveals that men are substantially more likely to be employed in full-time jobs, while women are considerably more represented in part-time roles and mini-jobs, typically characterised by fewer working hours and lower earnings. This confirms that women are more concentrated in precarious or non-standard employment. Moreover, women display a notably higher rate of inactivity.

However, it is important to underline that when focusing exclusively on asylum seekers (i.e., those who are still awaiting a decision – 326 cases), labour market conditions are inevitably less favourable: 36% of men and nearly 70% of women are either inactive or actively seeking employment. This outcome reflects the fact that those still awaiting a decision may be allowed to work but often face resistance from employers, who are required to invest in their training.

**Fig 11:**
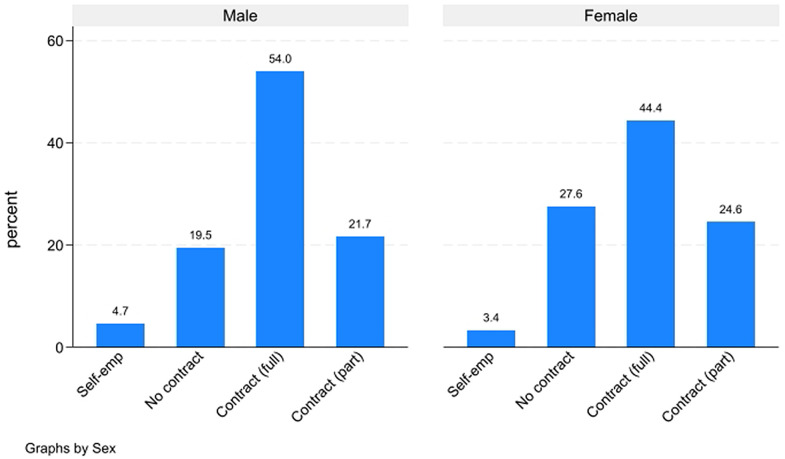
Type of employment contract (among workers and trainees).

Focusing only on workers and trainees (866 cases), the analysis of contractual arrangements (Fig 11) further confirms the gendered nature of labour market outcomes within our sample. Men are substantially more likely to hold a regular contract for all their working activities compared to women (54% versus 44%). Conversely, women show a higher incidence of employment without a formal contract (27.6% for women compared to 19.5% for men), highlighting their greater exposure to informal or precarious employment conditions. Additionally, self-employment appears marginal for both groups.

**Fig 12:**
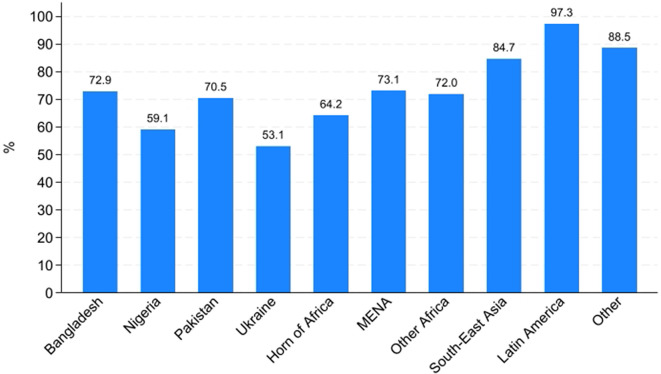
Percentage of workers by geographical origin. Men.

**Fig 13:**
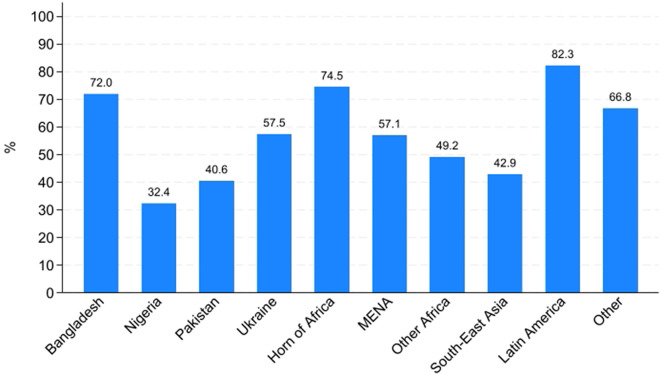
Percentage of workers by geographical origin. Women.

An analysis of AS&Rs’ employment rates by country of origin (Figs 12 and 13) reveals substantial differences both across countries and between men and women. Among men, employment rates are generally high, exceeding 70% in most countries, with particularly elevated figures for those from Latin America (97.3%), South-East Asia (84.7%) and Other European countries (88.5%). In contrast, males from Ukraine (53.1%) and Nigeria (59.1%) display notably lower employment rates.

For women (Fig 13), employment rates are consistently lower across nearly all areas of origin, confirming the gender gap identified in previous analyses. The most pronounced disparities between men and women are observed among those from Nigeria (59.1% for men vs. 32.4% for women) and from Other African countries (72% for men vs. 49.2% for women). Notably, women from Latin America (82.3%) and the Horn of Africa (74.5%) exhibit relatively high employment rates, even surpassing their male counterparts in the Horn of Africa (74.5% for women vs. 64.2% for men). However, women from Pakistan, Nigeria, and South-East Asia remain significantly underrepresented in the labour market, with employment rates well below 50%. In conclusion, these patterns underscore that labour market integration is related not only to gender but also to the area of origin. Cultural norms, co-ethnic networks, migration trajectories (including length of residence) and the degree of social acceptance by the host society likely contribute to characterising these cross-country and gender-specific variations.

### Social and material well-being

The assessment of the social and economic vulnerability of refugees and asylum seekers was assessed in *ItRAS* via two sets of indicators: the material and social deprivation scale and items addressing financial aspects of daily life.

The material and social deprivation scale [26] proves particularly useful when assessing individuals whose economic resources are difficult to quantify through conventional income measures. This is especially relevant for those engaged in multiple irregular or informal jobs over a year, for whom answering the question ‘How much income did you earn last year?’ may be inaccurate or even unfeasible. Additionally, some individuals rely on non-market exchanges or remit a significant portion of their income to their country of origin, meaning that reported earnings may not correspond to their actual standard of living. When comparing across nationalities, the ability to convert income into well-being is also connected with different structural conditions, access to services and cultural preferences. In contrast, material and social deprivation indicators offer a more direct assessment of people’s actual ability to afford goods and services that are essential to a decent standard of living.

This scale addresses both household-level and individual-level needs. Adhering to the official Eurostat construction threshold of “severe material and social deprivation”, we define severely deprived those experiencing an enforced lack of at least seven out of thirteen deprivation items.

Analysing the *ItRAS* data, refugees and asylum seekers were found to be more severely deprived than the general resident population in Italy: while only 5.9% of people residing in Italy are severely deprived, over 35% of the AS&Rs experience a lack of at least seven deprivation items. Among those severely deprived, 56.6% are men.

**Fig 14:**
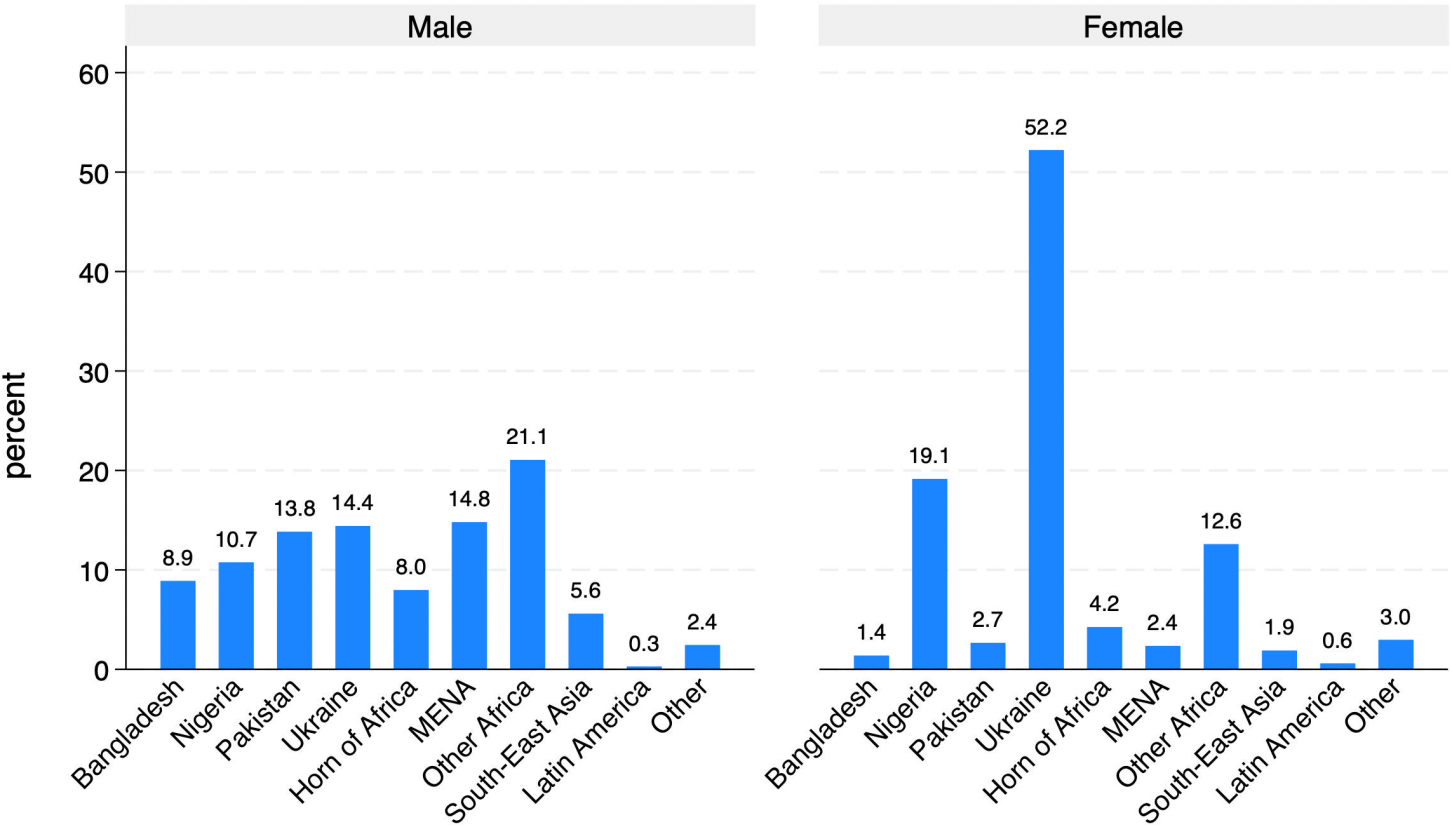
Origin distribution by sex for severely deprived refugees and asylum seekers (%).

As for the country or region of origin, people from Ukraine are more severely deprived with respect to the rest of AS&Rs - this might be due to their sudden displacement, making the scarcity of basic material resources harsher. People from Nigeria and “Other Africa” – as well as Pakistani refugees and asylum seekers – follow Ukrainians. The least affected seem to be Latin American people. When also taking gender into account, over 50% of Ukranian women are severely deprived, whereas only 0.6% of Latin-American women are; among men, only 0.3% of those coming from Latin America face severe deprivation, but over 20% of African men do. When also taking gender into account (Fig 14), over 50% of Ukranian women are severely deprived, whereas only 0.6% of Latin-American women are; among men, only 0.3% of those coming from Latin America face severe deprivation, but over 20% of African men do.

Contrary to what might be expected, almost 60% of severely deprived individuals (CI = 53.11%−64.43%) have been granted a high level of legal protection (i.e., refugee status or other form of international protection). Thus, refugees seem to fare worse than those with low legal protection (i.e., asylum seekers). This could be explained by the fact that the official reception system, which is in place while still waiting for status grant, ejects them from this safety net a few months after they are recognised as refugees. Once out of the system, their economic condition severely worsens, often forcing these people into homelessness and instability [18].

In addition to assessing the material deprivation, we also evaluated how much refugees and asylum seekers rely on subsidies – intended both as welfare-funded aids and community-leveraged funds. The results show that over 85% of respondents affirm that their living conditions would drastically worsen without their subsidies. Among these, more than 60% are women, probably due to increased barriers to the job market that make them rely more on monetary aid. Indeed, as mentioned earlier, even when employed, women’s position in the labour market is still more precarious than men’s.

**Fig 15:**
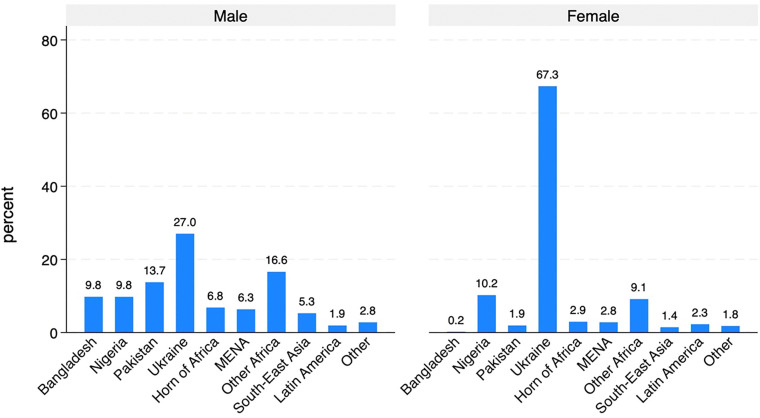
Sex and origin distribution for refugees and asylum seekers who heavily rely on subsidies (%).

As for the geographical origin, Ukrainian women – once again – stand out: in general, 50% of those relying on subsidies are, indeed, from this country; Latin-American people are also confirmed to fare much better than others. By gender, over 65% of Ukranian women rely heavily on subsidies, as well as 27% of Ukranian men; only 0.2% of Bangladeshi women and 1.9% of Latin-American men rely heavily on subsidies (Fig 15).

The same counter-intuitive result found for severe material deprivation is true for reliance on subsidies: among those heavily relying on subsidies, 76% were already granted asylum at the time of interview (CI = 71.57%−79.57%).

### Life in Italy

Finally, the *ItRAS* explores several other areas related to the process of integration in Italy, covering thematic areas, such as language command, opinion on the Italian lifestyle, perception and personal experiences of discrimination, satisfaction with current life, feeling of being welcome and migratory projects in the near future.

The integration process into a foreign country involves not only adapting to new rules and norms but also developing a sense of belonging and feeling welcomed by the host society. This also has an impact on the intention to stay in the hosting countries, to settle a new life, to plan for the future [67], develop new projects and invest in the new life in Italy.

Empirical studies have shown that life satisfaction among refugees is strongly influenced by the perceived level of acceptance in the host country as well as by its economic inequality [68]. Accordingly, discrimination is widely recognised as a crucial factor negatively impacting refugees’ physical and mental health, often resulting in the avoidance of healthcare services due to fear of ridicule or mistreatment [69]. Discriminatory experiences are also linked to social exclusion and barriers in accessing housing, education and employment [70].

**Fig 16:**
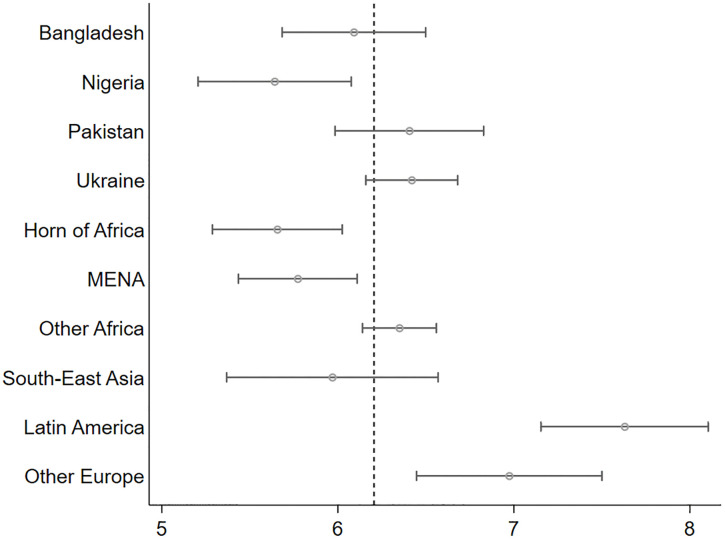
How much are you satisfied with your current life?. (means by geographical area of origin and CI 95%).

In the *ItRAS* survey, AS&Rs were asked whether they were satisfied with their current life. Their average level of life satisfaction is not particularly high (on average slightly above 6, on a scale from 1 to 10). Those from Latin America and European countries (with the exclusion of Ukrainians) stand significantly above the mean, while Africans (from Nigeria, Ethiopia, Eritrea and the MENA region) are definitely below (Fig 16).

**Fig 17:**
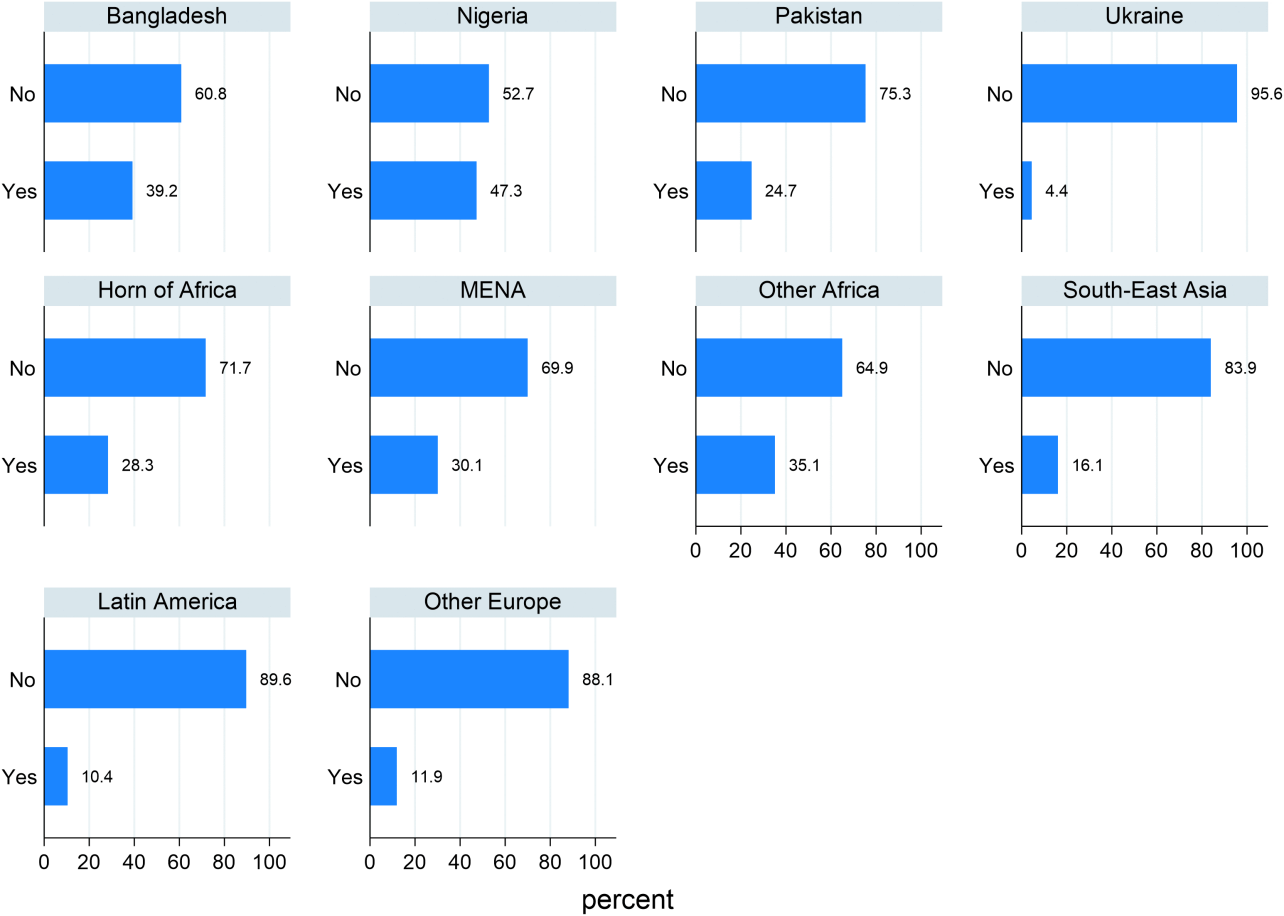
Differences by geographical area of origin in the experiences of harassment. (Yes: at least one experience in the last year; No: it never happened in the last year).

Findings from *ItRAS* also show that individuals from EU countries (which in our classification include the few stateless) and from Latin America are also more likely to report a strong sense of welcome and rarer experiences of harassment (Fig 17), compared to other AS&Rs. Overall, we observe that about 62% of AS&Rs feel welcome in Italy (very much or for the most part) and 72% want to stay in Italy.

Focusing on episodes of harassment, individuals from Africa report notably higher levels of harassment compared to those from other regions. Around 65–70% report never having experienced harassment, but Nigerian people point out as more exposed, with only 52.7% declaring not being the object of harassment. Asian respondents seem to be less exposed to this kind of discrimination: 75% of Pakistani and around 61% of Bangladeshi do not report any episode of harassment in the last year and people from Southeast Asia (mainly Afghans) are among the least exposed.

Similar results were found for the other forms of discrimination explored (less respect, poorer services, treated as less intelligent or as people to be afraid of).

**Fig 18:**
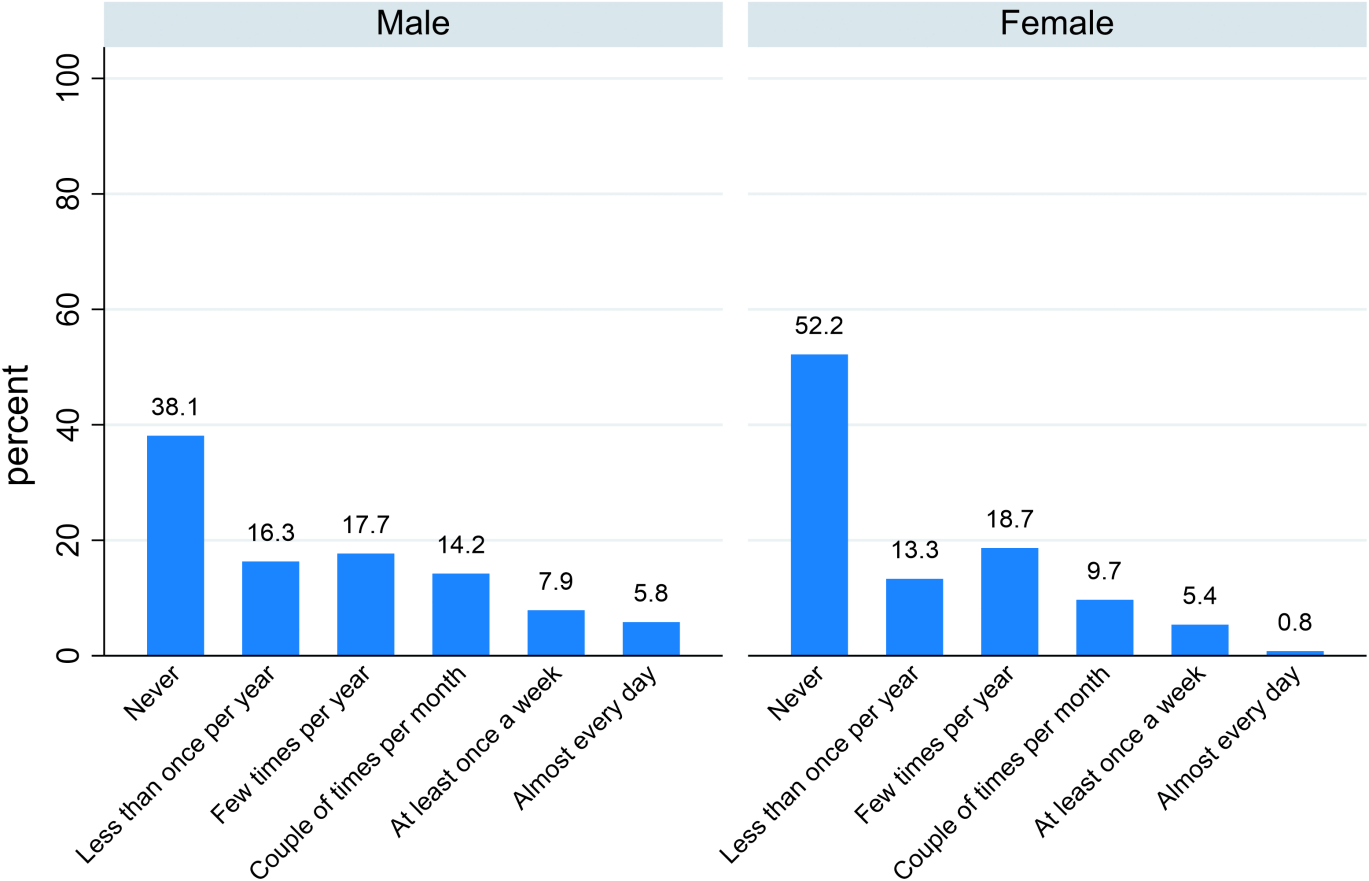
Gender differences in the frequency of experiences of lack of respect (%).

*ItRAS* highlights gender-based differences in the experiences of discrimination suffered in Italy by AS&Rs: women report experiencing it less frequently than men for every aspect of discrimination investigated. Particularly, Fig 18 reveals the most widespread form of discrimination suffered by AS&Rs, i.e., being treated with less respect than native Italians are. Particularly, about 48% of women and 62% of men experienced a lack of respect because of their being foreigners, with a different frequency (from less than once a year to almost every day).

**Fig 19:**
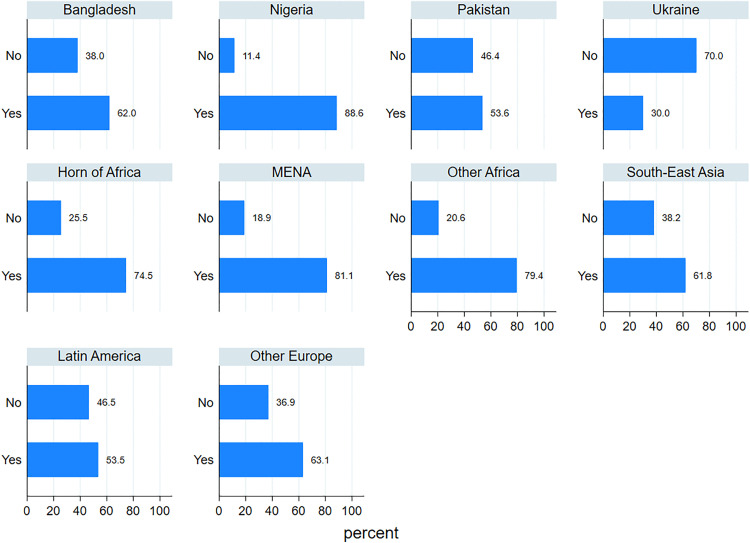
Differences by geographical area of origin in the experiences of a lack of respect. (Yes: at least one experience in the last year; No: it never happened over the last year).

Furthermore, Fig 19 highlights that respondents of African origin report among the highest frequencies of experiences of lack of respect, compared to other nationality groups, while Ukrainians seem to live a clearly better situation.

The quality of life in Italy and the perception of being welcome in the host country have a clear impact on the intentions of AS&Rs to remain in Italy [67]. Most refugees and asylum seekers in Italy appear to plan at least a medium-term stay, with only 14% indicating an intention to leave the country within the next 12 months. This share varies significantly by nationality (likely reflecting perceptions of the national context of origin): only 9% of Syrians intend to leave, compared to 20% of Ukrainians and 41% of Eritreans. Intentions also differ according to the type of residence permit. Among those holding refugee status, 15.8% plan to leave Italy within the next year, compared to 20% of Ukrainians under temporary protection and just 4.7% of individuals with a pending asylum application.

The legal and bureaucratic dimensions of settling in Italy often present significant challenges for AS&Rs; for many, accessing services and securing the recognition of their rights becomes a frustrating and seemingly endless struggle. The survey sheds light on the often lengthy and uncertain bureaucratic journey faced by asylum seekers in Italy.

In terms of integrating into the Italian education and healthcare systems, the recognition of prior educational qualifications represents another bureaucratic hurdle. Only 15.2% of *ItRAS* population reported initiating the process to have their qualifications officially recognised. Among these, 36.8% completed the procedure successfully, while 18.7% were still awaiting its conclusion. The process itself appears to be relatively efficient compared to asylum decisions, with an average duration of 5.7 months and a median of 3 months.

Despite these challenges, most respondents reported successful access to essential public services. A significant majority – 77.3% – are registered in the official population registry (*anagrafe*), which is often a prerequisite for accessing other services. Health coverage is also widespread, with 92.3% holding a national health insurance card and 83.7% having been assigned a general physician, indicating a relatively high degree of integration into the healthcare system.

## Discussion

The AVRAI project aimed to fill a significant gap in the empirical understanding of the living conditions, vulnerabilities and integration experiences of refugees and asylum seekers in Italy. In a context where the absence of dedicated data collection has long limited evidence-based policymaking [6,12], the AVRAI project and the *ItRAS* survey were designed to capture the complex realities faced by asylum seekers, refugees and beneficiaries of other forms of international protection. The survey is built through a rigorous, targeted sampling strategy and aligned with leading European surveys [8], offering unprecedented data covering multiple dimensions of AS&Rs’ vulnerability.

This paper aims at presenting the survey and some descriptive findings. Other outputs of the project will ground on these premises.

The findings so far focus on the multidimensional nature of AS&Rs integration and vulnerability. While most respondents benefit from protection-related status, precarious legal conditions remain widespread, especially among recent arrivals from Bangladesh, Pakistan and the MENA region. Gender disparities also emerge sharply, with women more represented among those with protection status but also facing unique vulnerabilities, consistent with findings by Phillimore [71] on gendered trajectories of integration.

Health indicators reflect similar complexities. While self-rated health appears relatively positive, consistent with broader “healthy migrant” effects [33], disparities by gender, country of origin and time of arrival are evident. Mental health outcomes are notably poorer. The findings point to marked disparities across gender, origin and length of stay; women, newcomers and those from Central and the Horn of Africa tend to report poorer outcomes consistently with [35,72]. Importantly, even among those residing in Italy for several years, unmet medical needs remain widespread, suggesting that the psychological toll of migration and forced displacement remains high and is insufficiently addressed. In addition to this, prolonged exposure to legal and economic insecurity and experiences of discrimination may gradually erode initial health advantages, such as in [69].

Economic vulnerability further compounds these challenges. Severe material deprivation affects over one-third of AS&Rs in Italy, a proportion dramatically exceeding that observed in the general Italian population. Employment outcomes are strongly gendered, with women facing greater barriers to stable and formal employment. Patterns of labour market integration differ by origin, reflecting variations in migration drivers and levels of human capital [59,60,73,74].

Food insecurity, a frequently overlooked dimension, emerges as a critical concern, particularly among respondents from Nigeria, the MENA region and Sub-Saharan Africa. These findings, consistent with international evidence [51,55], represent the first evidence in the Italian context.

Despite these challenges, resilience levels among AS&Rs remain generally high. Variations by education, gender and geographical origin align with results from multidimensional models of resilience that emphasise the role of both individual and contextual factors [69,75,76]. Higher educational attainment, in particular, appears to serve as a crucial protective factor, reinforcing the role of human capital in navigating adversity.

Experiences of life in Italy, however, remain ambivalent [77]. While many AS&Rs express satisfaction and a desire to stay, experiences of harassment and discrimination are frequent, especially among African-origin respondents. Perceptions of being welcomed also vary strongly by area of origin, supporting evidence of racism and xenophobia as barriers to full social inclusion [67,68]. It is, however, important to recall that Italy has historically been, and continues to be, largely a country of transit for many refugees, which might limit their prospects for stable integration and long-term settlement.

## Conclusions

Building on the detailed evidence gathered through the *ItRAS* survey, it is evident that Italy’s asylum framework – far from functioning as a neutral administrative mechanism – presents itself as a patchwork of fragmentation, delay, and, at times, opacity. Respondents reported average waiting times exceeding ten months and highlighted the psychological strain of prolonged legal uncertainty; these lived experiences illuminate the structural weaknesses underlying the Italian system.

The broader European migration debate remains deeply polarised – a dynamic that resonates in the ambivalent experiences recorded by the *ItRAS* survey. Although approximately 62% of AS&Rs report feeling welcome in Italy and 72% express a desire to remain, between 40% and 60% have suffered at least one incident of harassment over the past year. On the one hand, migrants, including also AS&Rs, are recognised as potentially bolstering economic productivity and addressing labour shortages in ageing societies; on the other hand, public discourse, infused with anxieties about national identity, social cohesion and the presumed overburdening of welfare services, has been co-opted by far-right movements to justify ever-tighter controls [22].

Although in our survey the majority of respondents declare at least a medium-term stay plan, Italy has a long‐standing role as a transit country, which compounds the polarised dynamics just exposed. Historically, and to this day, Italy is primarily viewed as a stepping‐stone toward other European destinations, a reality that both constrains prospects for stable integration and reinforces a sense of uncertainty among those who must navigate its reception system.

The current European asylum framework presents fundamental structural and procedural deficiencies. The Dublin III Regulation creates unequal responsibility distribution among Member States while limiting asylum seekers’ autonomy in choosing their destination country. Although the 2024 New Pact on Migration and Asylum proposes enhanced solidarity mechanisms, its implementation from 2026 raises concerns about persistent inequalities in protection access.

Additionally, Italy faces other structural weaknesses: the expansion of accelerated procedures which compromises essential procedural guarantees; a systematic underinvestment in administrative capacity and resources which cause a significant application backlog; bureaucratic obstacles, such as disproportionate documentary requirements. The growing preference for subsidiary protection over full refugee status reflects a restrictive policy orientation that prioritizes administrative efficiency and political considerations over comprehensive rights protection. Ultimately, these developments reveal an asylum system increasingly oriented toward distinguishing “deserving” from “undeserving” refugees, while struggling to reconcile legal obligations with contemporary migration complexities. In the complex Italian legal and operational framework, the *ItRAS* survey helps shedding light on the characteristics and experiences of AS&Rs, offering a vital empirical basis to guide targeted interventions that can transform fragmented procedures into coherent, rights-based pathways toward protection and long-term integration.

The findings presented in this paper, while only setting the stage for further in-depth analysis, already call for urgent differentiated and integrated policy responses. Targeted support is essential to address the legal insecurity, economic vulnerability and mental health risks disproportionately affecting certain subgroups, particularly women and those recently arrived. Strengthening early integration measures, enhancing access to employment, healthcare services and education and actively combating discrimination could help mitigate the emergence of groups of individuals experiencing persistent social exclusion and severe poverty to build sustainable and inclusive societies.
